# Recent Positive Selection Has Acted on Genes Encoding Proteins with More Interactions within the Whole Human Interactome

**DOI:** 10.1093/gbe/evv055

**Published:** 2015-04-02

**Authors:** Pierre Luisi, David Alvarez-Ponce, Marc Pybus, Mario A. Fares, Jaume Bertranpetit, Hafid Laayouni

**Affiliations:** ^1^Institute of Evolutionary Biology, Universitat Pompeu Fabra-CSIC, CEXS-UPF-PRBB, Barcelona, Catalonia, Spain; ^2^Integrative Systems Biology Group, Instituto de Biología Molecular y Celular de Plantas, Consejo Superior de Investigaciones Científicas (CSIC)-Universidad Politécnica de Valencia (UPV), Spain; ^3^Biology Department, University of Nevada, Reno; ^4^Smurfit Institute of Genetics, University of Dublin, Trinity College, Ireland; ^5^Departament de Genètica i de Microbiologia, Grup de Biologia Evolutiva (GBE), Universitat Autonòma de Barcelona, Bellaterra, Spain

**Keywords:** physical protein interaction, protein interaction network, natural selection, positive selection, mammals, humans

## Abstract

Genes vary in their likelihood to undergo adaptive evolution. The genomic factors that determine adaptability, however, remain poorly understood. Genes function in the context of molecular networks, with some occupying more important positions than others and thus being likely to be under stronger selective pressures. However, how positive selection distributes across the different parts of molecular networks is still not fully understood. Here, we inferred positive selection using comparative genomics and population genetics approaches through the comparison of 10 mammalian and 270 human genomes, respectively. In agreement with previous results, we found that genes with lower network centralities are more likely to evolve under positive selection (as inferred from divergence data). Surprisingly, polymorphism data yield results in the opposite direction than divergence data: Genes with higher centralities are more likely to have been targeted by recent positive selection during recent human evolution. Our results indicate that the relationship between centrality and the impact of adaptive evolution highly depends on the mode of positive selection and/or the evolutionary time-scale.

## Introduction

In recent years, the availability of large-scale network and genomic data sets has allowed researchers to study the relationship between the position of proteins within molecular networks and their patterns of molecular evolution (Cork and Purugganan 2004; Wagner 2012; Alvarez-Ponce 2014; Montanucci et al. 2014). These studies have shown that the strength of purifying selection acting on individual genes is affected by the position that their encoded products occupy in molecular networks. Indeed, genes acting at the centre of protein–protein interaction networks (PINs) and metabolic networks (i.e., genes coding for proteins with many interactions or connections) evolve under higher levels of purifying selection than those acting at the network periphery (Fraser et al. 2002; Hahn and Kern 2005; Vitkup et al. 2006; Alvarez-Ponce 2012; Alvarez-Ponce and Fares 2012) (but see Jordan et al. 2003; Hahn et al. 2004). Furthermore, interacting proteins evolve at similar rates, probably as a result of molecular coevolution (Fraser et al. 2002; Agrafioti et al. 2005; Codoñer and Fares 2008; Cui et al. 2009; Lovell and Robertson 2010; Pérez-Bercoff et al. 2013).

Less well understood, however, is how adaptive events distribute across molecular pathways and networks. Some evidence supports that adaptive events tend to occur in less centrally located regions of gene networks. In an early study using two genomes, the human and chimpanzee genomes, Kim et al. (2007) found that positive selection often targeted genes acting at the periphery of the PIN. Powerful detection of positive selection requires, nevertheless, comparing many genomes (Anisimova et al. 2002; Kosiol et al. 2008), making it appropriate to reevaluate this trend in light of the currently available mammalian genomes.

In addition, recent population genetics studies of certain metabolic and signaling pathways appear to contradict the notion that positive selection targets preferentially the periphery of molecular networks. Indeed, positive selection often targets genes acting at the most “influential” positions of these pathways, including the most central genes in the human insulin/mammalian Target of Rapamycin pathway (Luisi et al. 2012), genes acting at bifurcation points of the human N-glycosylation pathway (Dall’Olio et al. 2012) and the *Drosophila* pathways involved in glucose metabolism (Flowers et al. 2007), and the gene encoding the first enzyme of the *Arabidopsis* glucosinolate pathway (Olson-Manning et al. 2013). Simulation studies also indicate that adaptation preferentially targets genes acting at the upstream and branch-point parts of pathways, at least when the system is far from the fitness optimum (Wright and Rausher 2010; Rausher 2012). Proteins occupying these key network positions are expected to exert strong influence over the pathway function, and thus on the associated phenotypes and organism’s fitness (Wright and Rausher 2010; Rausher 2012; Olson-Manning et al. 2013). Therefore, positive selection on genes encoding such proteins may lead to rapid adaptation.

Here, we make use of the unprecedented wealth of genomic (1000 Genomes Project Consortium 2012; Kersey et al. 2012) and interactomic data (Stark et al. 2011) to ascertain what parts of the human PIN were affected by positive selection, using both comparative genomics and population genetics approaches. We found that positive selection, as inferred from divergence data, preferentially targets genes acting at more peripheral positions in the network, in agreement with previous observations (Kim et al. 2007). Conversely, genes with signatures of recent positive selection, identified considering polymorphism data, occupy more central parts of the network. We discuss on the apparently contradictory results from divergence and polymorphism data and propose an evolutionary scenario reconciling both patterns.

## Materials and Methods

### Reconstructing the Human PIN

The human PIN was reconstructed from the interactions available from the BioGRID database version 3.1.81 (Stark et al. 2011). Only nonredundant physical interactions were considered to calculate centrality measures. We removed from our analysis proteins without an Ensembl ID as well as Ubiquitin C (encoded by the gene with Ensembl ID ENSG00000150991), which has an outlier degree centrality. For each protein, degree was computed as the total number of interactions in which it is involved, and betweenness and closeness centralities were computed using the NetworkX Python library (https://networkx.github.io/).

### Inferring Natural Selection from Ten Mammalian Genomes

In order to infer events of positive selection that have occurred during the evolution of mammals we used sequence data for a set of mammals, enriched in primates. The analysis was restricted to ten high-coverage genomes: Human, chimpanzee, gorilla, orangutan, macaque, mouse, rat, cow, dog, and opossum. The platypus genome was not included in the analysis, as the currently available assembly is highly fragmented, making gene annotation difficult. Also excluded were nonmammalian genomes, in order to avoid the problem of saturation of synonymous sites (Smith JM and Smith NH 1996), and to maximize the number of genes with 1:1 orthologs in all studied genomes.

All protein and coding sequences (CDSs) for the selected genomes were obtained from Ensembl release 62 (Kersey et al. 2012). For each of the 9,041 human protein-coding genes represented in the PIN, we searched the nine nonhuman genomes for 1:1 orthologs using the best reciprocal BLAST (Basic Local Alignment Search Tool) approach. First, we selected the longest protein (or, in the case of multiple proteins sharing the maximal length, that classified as the canonical isoform), and used it as query in a BLASTP search against each of the nonhuman proteomes. Second, for the best hit in each proteome, we performed a BLASTP search against the human proteome. If the hit obtained in the second search was the original human protein, then it was considered to be a 1:1 ortholog. Only human genes with 1:1 orthologs in all nine nonhuman genomes were used in subsequent analyses (in total, 5,916 genes met this criterion).

Each group of orthologous proteins was aligned using ProbCons 1.12 (Do et al. 2005). Because tests of positive selection are sensitive to sequencing, annotation and alignment errors (Talavera and Castresana 2007; Scheinfeldt et al. 2009), we used highly stringent criteria to filter our alignments. First, unreliably aligned regions were removed using Gblocks version 0.91 b (Talavera and Castresana 2007), with default parameters. Additionally, we used an ad-hoc filtering procedure in order to remove annotation errors, including the following steps: 1) Identification of unique amino acid replacement (i.e., amino acids that are unique to a given species in a certain alignment column); 2) identification of alignment regions with a very high incidence of unique substitutions in the same species; in particular, we used a sliding window approach to identify regions of 15 amino acids containing ten or more unique substitutions in the same sequence, as well as regions of five amino acids containing five unique substitutions in the same sequence; these patterns are unlikely to represent true divergence between species, provided that the species included in the current analysis are relatively closely related; and 3) removal of these alignment regions. These procedures resulted in the removal of 35.5% of amino acid positions. The resulting filtered protein alignments were used to guide the alignment of the corresponding CDSs using an in-house BioPerl script.

We evaluated the impact of both purifying and positive selection on each orthologous group using the program codeml from the package PAML 4.4 (Yang et al. 2005). For each CDS alignment, three different evolutionary models (M0, M7, and M8) were fitted. First, for each gene, an overall nonsynonymous to synonymous divergence ratio (ω = *d*_N_/*d*_S_) estimate was obtained from the M0 model, which assumes a homogeneous ω for all branches in the tree and all codons in the alignment. This ratio was used as a proxy of the impact of purifying selection, with values of ω close to 0 indicating strong purifying selection, and values close to 1 indicating weak purifying selection. Second, in order to infer the action of positive selection, we applied the M7 versus M8 test (Nielsen and Yang 1998). The M7 model assumes that codons’ ω values follow a beta distribution, limited to the interval (0, 1), whereas model M8 allows for an additional class of codons with ω > 1. The likelihood ratio test was used to contrast whether model M8 fits the data significantly better than model M7. Twice the difference between the log-likelihoods of both nested models, [2Δ*ℓ* = 2 × (*ℓ*_M8_ − *ℓ*_M7_), where *ℓ_i_* is the log-likelihood of the observed data under model *i*], is assumed to follow a χ^2^ distribution with 2 degrees of freedom. In order to avoid the problem of local optima, for each gene each model was fitted three times, using different starting ω values (0.04, 0.4, and 4), and the computation with the highest likelihood was retained. The commonly accepted tree topology was used.

In order to discard potential alignment errors, not detected by our stringent filtering, the alignments corresponding to genes with *P* < 0.1 in the likelihood ratio test for positive selection were inspected visually. Alignment regions containing evident errors were manually removed using BioEdit v7.0.5.2 (Hall 1999), and analyses of positive selection were rerun. We obtained a list of 554 genes with putative signatures of positive selection (divPSGs; *P* < 0.05).

We also repeated the analysis of positive selection by considering two alternative alignment sets: 1) A set of human genes with 1:1 orthologs in three to nine nonhuman genomes (8,697 genes met this criterion) to which we applied the filtering process described above and 2) the set of 5,916 human genes with 1:1 orthologs in all nine nonhuman genomes without applying any alignment filtering.

### Inferring Natural Selection from 270 Human Genomes

We obtained phased genotypes from low-coverage data of the phase I of the 1000 Genomes Project (1000 Genomes Project Consortium 2012), which makes available data for over 36 million Single Nucleotide Variants (SNVs) for 1,092 individuals sampled from 14 populations worldwide. We used a subset of 270 individuals from YRI, CEU, and CHB populations. We focused on those three populations because they are representative of the human genetic diversity in three main geographic regions (Africa, West Eurasia, and East Asia) and signals of positive selection have been described to be extensively shared in related populations (Coop et al. 2009). Samples from American populations present a high level of admixture (1000 Genomes Project Consortium 2012), making difficult an accurate study of natural selection in these populations.

For each of the 9,041 genes contained in the PIN, we analyzed the genomic region corresponding to the transcript spanning the longest chromosome region. Gene coordinates were obtained from the release 37 of the human genome at NCBI (Flicek et al. 2010). We removed 365 genes located at sex chromosomes because some of the methods used to detect signals of positive selection have been devised for autosomal regions, or provide results that cannot be compared between genes located at autosomal and sex chromosomes. In order to increase the statistical power in the detection of positive selection, we removed from the analyses 96 genes with less than ten SNVs annotated in the 1000 Genomes Project.

We used the genetic map provided by the 1000 Genomes Consortium. Ancestral states inferred from comparison with orthologous sequences in the chimpanzee and rhesus macaque genomes were obtained from the UCSC Genome Bioinformatics Site (Karolchik et al. 2009) (http://genome.ucsc.edu/; table “snp128OrthoPanTro2RheMac2”).

Retained genes (a total of 8,580) have a length ranging from 0.414 to 2,305 kb (mean = 61.70 kb; median = 25.95 kb) and are covered by a total of 6,815,879 SNVs. The number of SNVs located in a gene ranges from 10 (28 genes) to 45,577, with a mean of 794.4 and a median of 312.

To identify the genes belonging to the PIN that have evolved under positive selection during human evolution, we applied three different tests: 1) The integrated Haplotype Score (Voight et al. 2006) (iHS), which aims to detect extended haplotype homozygosity (EHH) from the local haplotype structure; 2) the Cross-Population Composite Likelihood Ratio (Chen et al. 2010) (XP-CLR) method, based on the multilocus allele frequency differentiation between two populations; and 3) *DH* (Zeng et al. 2007), based on the excess of rare variants, which combines Tajima’s *D *(Tajima 1989) and Fay and Wu’s *H *(Fay and Wu 2000)*. *These tests are designed assuming the hard sweep model which states that a new advantageous mutation arises in the population and rapidly increases in frequency hitchhiking the surrounding neutral variants located on the same haplotype.

We computed a raw iHS for each SNV with ancestral state information following the method proposed by Voight et al. (2006). We used the script available at http://hgdp.uchicago.edu/Software/, which we slightly modified in order to speed up computation times; thresholds for EHH decay were modified from 0.25 to 0.15 and we used a size for the analyzed region of 0.2 Mb (original size: 2.5 Mb). We validated that these changes were previously described not to affect the sensitivity and specificity of the method through coalescent simulations (Pybus et al. 2014). Standardized iHS scores were obtained by grouping SNVs into 20 bins separated by a derived allele frequency (DAF) of 0.05, subtracting the mean, and dividing by the standard deviation for all SNVs in the same bin as in Voight et al. (2006). Extreme positive or negative values indicate high EHH of haplotypes carrying the ancestral or derived allele, respectively. Hence, we consider both extreme positive or negative iHS as potential signatures of positive selection. We integrated the |iHS| scores observed at each gene of interest into a gene-level summary statistic using the mean.

The XP-CLR method aims at detecting important genetic differentiation in an extended genomic region in comparison with a reference population. This method provides a good localization of the position of the selected variant (Chen et al. 2010). XP-CLR scores were computed at regularly spaced grid points (every 2 kb) using the information from SNVs within a flanking window of 0.2 cM. To account for different SNV densities among genomic regions, we restricted to 200 the maximal number of SNVs used to calculate XP-CLR scores within each window, by randomly removing SNVs in excess. We integrated the XP-CLR scores observed at each gene of interest into a gene-level summary statistic using the mean.

Extreme iHS and XP-CLR scores could also be attributable to the action of nonselective events, such as demographic changes and genetic drift. However, these selectively neutral events act randomly on the genome, in contrast with positive selection, which targets specific genes. Therefore, we adopted an outlier approach to infer the action of positive selection on PIN genes (Kelley et al. 2006; Teshima et al. 2006): We evaluated the significance of the scores for each gene by taking into account the whole genome context. For that purpose, we used a genomic gene-level background containing all annotated genes that were distant one from each other and from the 8,580 genes included in the analysis, by at least 5 kb and contained at least ten SNVs. The complete background gene set obtained thus includes 13,388 genomic regions and 8,431,716 SNVs. For each of these background genomic regions, we computed the mean summary statistics based on iHS and XP-CLR and then obtained gene-level empirical distributions. Empirical *P* values associated with iHS and XP-CLR for PIN genes were obtained using the gene-level score distributions obtained from the 13,388 genes in the background genome set.

For each gene, using the SNVs with ancestral state information, we also computed Tajima’s *D*, Fay and Wu’s *H* and *DH*, using a program kindly provided by Kai Zeng. For each gene, the *DH P* value was obtained as in Zeng et al. (2007) from Tajima’s *D* and Fay and Wu’s *H *by a bivariate comparison to their neutral distributions. However, instead of using 10,000 replicates of coalescent simulations to build these neutral distributions as in the original article, we used the 13,388 genomic regions described above in order to better take into account the demographic forces that acted on the studied populations.

In order to summarize the results of the three different tests, we combined the gene-level empirical *P* values obtained as described above using the Fisher combination test:

$$Z_{\text{F}}=-2\log\overset{i=3}{\underset{\text{i=1}}{\sum }}P_{\text{i}}.$$

where *P_i_* are the empirical *P* values obtained from the three tests. Thus, for each gene we obtained a unique *Z*_F_ score, which follows a χ^2^ distribution with 6 degrees of freedom. This combination requires independence of the three combined *P* values. We confirmed that deviation from this assumption would not affect our results (supplementary fig. S1, Supplementary Material online). We invoked positive selection if the *P* value associated with the *Z*_F_ score was below 5%. Therefore, we obtained four lists of genes with putative signatures of positive selection inferred from polymorphism data (polyPSGs): 3 populations + global level.

The major limitation of the methods implemented to detect positive selection using polymorphism data is that demographic events, such as population growth, bottleneck, and/or subdivision, can mimic patterns similar to those produced by selection. However, the outlier approach framework that we implemented and that combines three tests that consider three different molecular patterns (namely genetic differentiation, site frequency spectrum, and linkage disequilibrium) is very likely to overcome this issue.

In order to estimate the strength of purifying selection acting on the genes involved in the PIN, we calculated the average DAF among the 270 individuals belonging to YRI, CEU, and CHB populations (1000 Genomes Project Consortium 2012).

### Inferring Natural Selection using the McDonald and Kreitman Test

For each gene, we computed the polarized McDonald–Kreitman test (MK test; McDonald and Kreitman 1991) in order to infer the impact of natural selection in the human lineage, that is, since the split with chimpanzee. For that purpose, we defined nonsynonymous and synonymous sites as the 0- and 4-fold degenerated sites using the longest transcript for each of the 9,041 genes in the PIN. We then calculated the number of polymorphic nonsynonymous and synonymous sites (*P*_N_ and *P*_S_, respectively) in any of the three studied human populations (YRI, CEU, and CHB). We also estimated the number of nonsynonymous and synonymous substitutions (*D*_N_ and *D*_S_, respectively) that occurred in the human lineage by comparing the human and chimpanzee reference genomes using as outgroup the gorilla species: A substitution was assumed to have occurred in the human lineage when a site was different in the human sequence as compared with both chimpanzee and gorilla. We then estimated the Neutrality Index (NI) as

NI=PN/PSDN/DS.

We applied the Haldane’s correction for the NI whenever one of the four numbers (*P*_N_, *P*_S_, *D*_N__,_ or *D*_S_) was equal to 0 as follows:

NIcorrected=(PN+0.5)/(PS+0.5)(DN+0.5)/(DS+0.5).

We also tested for positive selection using a Fisher exact test performed on the contingency table containing the number of fixed substitutions and polymorphic sites for both nonsynonymous and synonymous positions.

We finally obtained an NI score and a *P* value from the Fisher’s exact test for 3,381 genes (those with more than three nonsynonymous and synonymous polymorphic sites and more than three nonsynonymous and synonymous substitutions).

### Analyzing DAF Patterns for Three Site Classes Nearby Genes under Positive Selection

In order to gain some insight into the functional nature of the variants targeted by recent positive selection in humans, we analyzed how extreme was the DAF observed at three site classes nearby polyPSGs: *cis-*eQTLs, nonsynonymous and synonymous variants. We retrieved expression Quantitative Trait Loci (eQTLs) annotations from two data sets which report eQTLs detected in different lymphoblastoid cell line samples: 1) “GEUVADIS” for 373 European samples from the 1000 Genomes Consortium (Lappalainen et al. 2013); and 2) “Liang et al.” for two British sample sets: MRCE (Morar et al. 2007) and MRCA (Dixon et al. 2007) analyzed together (Liang et al. 2013). We restricted our analyses to *cis-*eQTLs located within 100 kb of the associated gene. We then identified the 0- and 4-fold degenerated sites for all transcripts of the PIN genes using Ensembl release 65. We removed all sites whose classification as 0- or 4-fold degenerated depended on the transcript considered. The remaining 0- and 4-fold degenerated sites were considered as nonsynonymous and synonymous sites, respectively.

For each gene and site class, we calculated the maximum DAF observed in the CEU population. Genes for which the DAF score was missing for at least one of the three site classes were removed from the analysis. We obtained a maximum DAF score for the three site classes for 358 and 198 PIN genes when using the “GEUVADIS” and “Liang et al.” eQTL annotations. Among them, 29 and 14 genes exhibit a signal of recent positive selection as inferred from polymorphism data (polyPSGs).

For each site class, we contrasted whether polyPSGs show a higher median of the maximum DAF scores through 10,000 random permutations from the PIN genes.

We obtained a maximum DAF score for all three site classes for only two and six polyPSGs when using eQTLs detected in YRI samples in the studies performed by Lappalainen et al. (2013) and Pickrell et al. (2010), respectively, limiting the power of this analysis in the YRI population.

### Determining Fitness Effects of Genes

Using data from the Mouse Genome Database (Bult et al. 2008) (“MRK_Ensembl_Pheno.rpt” file downloaded on October 7, 2010), we classified genes as essential and nonessential when described to be lethal and viable when knocked out in mice, respectively. We retrieved such information for 3,994 genes represented in the PIN. However, given that essentiality may evolve relatively fast (Zhang and He 2005), essential genes in mouse may not be essential in humans.

Therefore, we also used the functional indispensability score (Khurana et al. 2013) estimated from functional and evolutionary properties. This score accurately distinguishes between essential genes (those showing clinical features of death before puberty or infertility when Loss-of-Function—LoF—mutations occur; Liao and Zhang 2008) and LoF-tolerant genes (those observed to contain homozygous LoF mutations in at least one individual in the 1000 Genomes Pilot Data; MacArthur et al. 2012). We obtained the functional indispensability score for 8,816 genes involved in the PIN.

## Results

### Positive selection inferred from divergence data and gene centrality in the human PIN.

We used ten mammalian genomes (Kersey et al. 2012) to infer events of positive selection that took place within the last approximately 165 Myr. Only genes with 1:1 orthologs in all ten species were used, and sequence alignments were stringently filtered prior to our analyses (see Materials and Methods). The test used in this study looks for a nonsynonymous to synonymous divergence ratio (ω = *d*_N_/*d*_S_) higher than 1 at a subset of codons (Nielsen and Yang 1998). It is based on a positive selection likelihood score, termed 2Δ*ℓ* (see Materials and Methods), that is proportional to the likelihood of positive selection*. *We identified a total of 554 putative positively selected genes (divPSGs; those with *P* < 0.05).

**Fig. 1.—:**
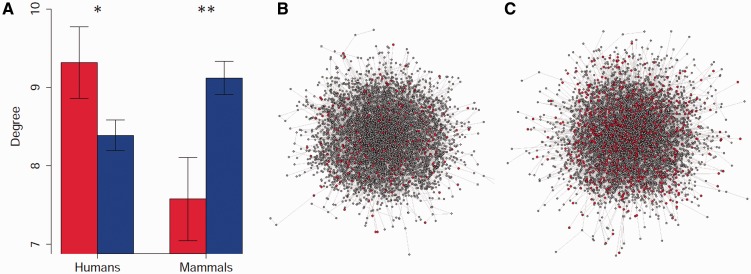
Distribution of genes with putative signatures of positive selection within the PIN. *Z*_F_ and 2Δ*ℓ *were used to estimate the likelihood of having evolved under positive selection in human populations and in mammals, respectively*.* (*A*) Average degrees (number of interactions) for genes with and without signatures of positive selection. We represent the mean of centrality measure ± 1 SE for the genes with a putative signal of positive selection (in red) and the other genes (in blue). The significance of the differences between the mean of both groups was assessed through 10,000 permutations. Asterisks represent significant differences. *P < 0.05; **P < 0.01. (*B*) Human PIN with genes with signatures of positive selection according to divergence data (*P* < 0.05 estimated from 2Δ*ℓ*) represented in red. (*C*) Human PIN with genes with signatures of positive selection according to polymorphism data represented in red.

We measured the difference in the mean degree (number of protein–protein interactions, or number of proteins with which a protein interacts) between divPSGs and the other genes in the network (non-divPSGs), and tested whether this difference was expected at random through 10,000 random permutations of the two groups containing divPSGs and non-divPSGs. We observed that divPSGs encode proteins with a significantly lower degree than non-divPSGs (permutation test: *P = *0.0067; fig. 1*A*; supplementary table S1, Supplementary Material online). Indeed, divPSGs and non-divPSGs encode proteins with, on average, 7.587 and 9.122 interactions, respectively, that is, the degree for divPSGs is 17% lower than the one observed for non-divPSGs. The magnitude of this difference is similar to that previously described (Kim et al. 2007).

**Fig. 2.—:**
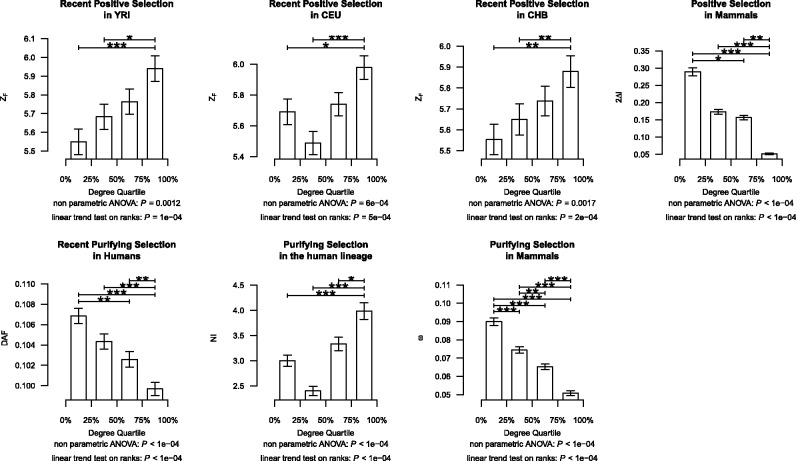
Impact of natural selection among groups of genes divided according to degree quartiles. Genes were divided into four groups according to the degree quartiles. The median selection score ± 1 median absolute deviation for each group is represented in the *y* axis. *Z*_F_ and 2Δ*ℓ *scores were used to estimate the likelihood of positive selection in human populations and in mammals, respectively*.* DAF, NI, and ω were used to estimate the impact of purifying selection in recent human populations, in the human lineage, and in mammals, respectively. Lower DAF and ω indicate higher evolutionary constraint estimated from polymorphism and divergence data, respectively, whereas higher NI scores indicate higher evolutionary constraint estimated from both polymorphism and divergence data. A nonparametric ANOVA analysis was performed to contrast whether the medians of the scores are equal across the groups. A trend test on ranks was also carried out to test for a linear relationship between the four groups (encoded from 1 to 4) and natural selection scores. A Tukey’s honestly significant difference test was further applied to test for all pairwise differences. Significantly different pairs are marked with asterisks according to the level of significance. *P < 0.05; **P < 0.01; ***P < 0.001.

We next observed that log-likelihood increments (2Δ*ℓ* scores) from the positive selection test exhibit a significant negative correlation with proteins’ degrees (Spearman’s rank correlation coefficient, *ρ* = −0.0841; *P < *0.0001; table 1), indicating that central genes are less likely to be under positive selection. Finally, when proteins were binned into four degree classes (low, medium-low, medium-high, and high degree) according to the first, second, and third quartiles, we observed a continuous decrease in their positive selection likelihood scores (2Δ*ℓ*) (fig. 2*D*; table 1). Indeed, the nonparametric analysis of variance (ANOVA) *F-*test is significant (*P < *0.0001), and there is a trend toward higher 2Δ*ℓ* scores in the lower degree groups (linear trend test on ranks; *P < *0.0001). We validated those results using two alternative alignment sets: One using unfiltered alignments for genes present in all ten species, and another using filtered genes with 1:1 orthologs in 4–10 species (supplementary note, fig. S2, and table S2, Supplementary Material online).

**Table 1:** Relationship between Degree and the Impact of Natural Selection

		Positive Selection				Purifying Selection		
		YRI (*Z*_F_)	CEU (*Z*_F_)	CHB (*Z*_F_)	Mammals (2Δ*ℓ*)	Recent Humans (DAF)	Humans (NI)	Mammals (ω)
Spearman correlation^a^	*Ρ*	0.0501	0.0409	0.0471	−0.0841	−0.0879	0.0770	−0.2039
	*P* value	1.11 × 10^−5^***	0.0004***	3.48 × 10^−5^***	9.29 × 10^−11^***	4.51 × 10^−16^***	7.29 × 10^−06^***	6.91 × 10^−56^***
Partial Spearman correlation^b^	*ρ*	0.0451	0.0326	0.0374	−0.0340	−0.0668	0.0314	−0.1698
	*P* value	0.0001***	0.0059**	0.0015**	0.0107*	2.35 × 10^−09^***	0.0742	2.79 × 10^−37^***
Nonparametric ANOVA^c^	*F*	5.324	5.844	5.074	11.90	18.03	9.084	77.85
	*P* value	0.0012**	0.0006***	0.0017**	9.18 × 10^−08^***	1.16 × 10^−11^***	5.49 × 10^−06^***	2.26 × 10^−49^***
Trend test on ranks^c^	*F*	15.88	12.14	14.12	33.51	52.60	20.66	229.4
	*P* value	6.79 × 10^−5^***	0.0005**	0.0002***	7.45 × 10^−09^***	4.43 × 10^−13^***	5.67 × 10^−06^***	7.30 × 10^−51^***
Partial nonparametric ANOVA^b^^,^^c^	*F*	2.731	3.149	2.080	2.537	6.353	2.343	51.93
	*P* value	0.0423*	0.0240*	0.1006	0.0548	0.0003***	0.0713	4.27 × 10^−33^***
Partial trend test on ranks^b^^,^^c^	*F*	7.794	2.360	5.107	6.281	16.48	2.964	153.6
	*P* value	0.0053**	0.1246	0.0239*	0.0122*	4.97 × 10^−5^***	0.0852	8.05 × 10^−35^***

^a^Spearman correlation between degree and selection scores (*Z*_F_ for positive selection in YRI, CEU, and CHB populations; 2Δ*ℓ *for positive selection in mammals; DAF for purifying selection during recent human evolution; NI for purifying selection in the human lineage; and ω for purifying selection in mammals). High *Z*_F _and 2Δ*ℓ *scores indicate a higher probability of having evolved under positive selection as inferred from polymorphism and divergence data, respectively. Low DAF and ω scores indicate higher evolutionary constraint estimated from polymorphism and divergence data, respectively, whereas high NI scores indicate higher evolutionary constraint estimated from both polymorphism and divergence data.

^b^In order to test for an association between degree and natural selection scores while controlling for putatively confounding factors, we applied a linear regression between the selection scores and protein length, expression level and breadth. The linear regression residuals were then used to perform the Spearman’s correlation analysis, the nonparametric ANOVA, and the linear trend on ranks test.

^c^Nonparametric ANOVA and linear trend tests on ranks performed to contrast whether the score used as a proxy of natural selection are equal across the degree groups.

*P < 0.05; **P < 0.01; ***P < 0.001.

Taken together, our observations indicate that adaptation (as inferred from divergence data) more frequently occurs at the less connected proteins of the human interactome, consistent with previous observations (Kim et al. 2007).

### Positive selection inferred from polymorphism data and gene centrality in the human PIN.

We inferred recent events of positive selection in humans using genomic data from three different populations: Yoruba in Nigeria (YRI), Northern European ancestry sampled in Utah (CEU) and Han Chinese in Beijing (CHB). We used a Fisher’s combination (*Z*_F_ score) of three tests of positive selection assuming the hard sweep model: XP-CLR (Chen et al. 2010), iHS (Voight et al. 2006), and *DH* (Zeng et al. 2007) (see Materials and Methods). Assuming that *Z*_F_ follows a χ^2^ distribution with 6 degrees of freedom, we identified putative positively selected genes (polyPSGs).

We measured the difference in the mean degree between these genes and genes without evidences of having evolved under positive selection (non-polyPSGs) (fig. 1*A* and supplementary table S1, Supplementary Material online). When all populations were analyzed together (global analysis), we observed a statistically significant higher degree for genes with signatures of positive selection (permutation test: *P = *0.0254)*.* Indeed, polyPSGs and non-polyPSGs encode proteins with, on average, 9.637 and 8.107 interactions, respectively, that is, the degree for polyPSGs is 19% higher than that observed for non-polyPSGs. The magnitude of this difference is similar to that observed at the interspecific level, yet in the opposite direction. When the three populations were considered separately, polyPSGs were always more connected than non-polyPSGs, although the test was significant only for YRI (supplementary table S1, Supplementary Material online).

*Z*_F_ scores and network degrees exhibit a significant positive correlation for all three populations (table 1). Finally, comparison of *Z*_F_ scores for the four degree groups based on degree quartiles (low, medium-low, medium-high, and high degree) using a nonparametric ANOVA showed significant differences in all three populations, as a result of higher *Z*_F_ scores at the highest degree groups, according to a linear trend test on ranks (fig. 2*A–C*; table 1). These results were reproduced using the three positive selection statistics separately (*DH, *iHS and XP-CLR in all populations, except XP-CLR in CEU and CHB), and also using the Composite of Multiple Signals method (Grossman et al. 2013, 2010) (supplementary note, fig. S3, and table S3, Supplementary Material online). Furthermore, the observed trends remain significant when removing the putative effect of linkage disequilibrium among genes by using a subset of unlinked genes (see supplementary note, fig. S4, and table S4, Supplementary Material online).

These analyses indicate that genes encoding proteins with a greater number of interactions in the human PIN are more likely to present signals of recent selective sweeps than those acting at more peripheral positions.

### Positive selection inferred from polymorphism and divergence data in the human PIN

We inferred positive selection in the human lineage applying the polarized MK test (McDonald and Kreitman 1991) on genomic data from three different populations (YRI, CEU, and CHB) along with three reference genomes (human, chimpanzee, and gorilla). As the genetic diversity in the human lineage and the human–chimp divergence are reduced, the MK test is not sensitive enough to detect selective events that occurred during the evolution of the human lineage (Zhai et al. 2009). Indeed, we obtained significant *P* values (at a significance level of 5%) for only four genes in the PIN, making difficult an accurate network-level analysis for positive selection at this evolutionary time-scale. A more powerful alternative to the Fisher’s exact test on the proportion of synonymous and nonsynonymous variants that are fixed between species or segregating in the lineage of interest is to contrast whether the parameter γ of the Poisson random field model is negative using a maximum-likelihood framework (Sawyer and Hartl 1992). We therefore downloaded the results from a previous implementation of the MK test between human and chimpanzee following this framework (Bustamante et al. 2005). In this study, however, the authors did not polarize the test (using an outgroup species), which would have allowed detecting putative selective events in specific lineages. We obtained a *P* value for 3,077 genes in the PIN, of which 210 genes exhibited a signal of selection (*P < *0.05). Genes under positive selection exhibit a higher degree centrality (average: 8.162 interactions) than the other genes (average degree: 7.481). However, the difference is not significant according to 10,000 permutations (*P = *0.232).

As the data set obtained from Bustamante et al. (2005) does not allow to study positive selection specifically in the human lineage, we decided not to use it for further analyses described below. We rather used the Neutral Index from our own implementation of the polarized-MK test as an estimate of the strength of purifying selection during human evolution.

### Correcting for Several Putative Confounding Factors and Validations

A number of factors correlate with both network centrality and the likelihood of observing positive selection, and might thus be confounding our observations. In order to discard this possibility, we conducted a number of validations.

In agreement with previous results (Fraser et al. 2002; Hahn and Kern 2005; Vitkup et al. 2006; Alvarez-Ponce 2012; Alvarez-Ponce and Fares 2012), we observed that purifying selection is stronger in genes acting at the centre of the human PIN than at those acting at the periphery, regardless of whether it was measured from the ω ratio, the NI or the DAF (fig. 2*E–G* and table 1). Purifying selection, through background selection (BGS), can produce signatures that can be confounded with positive selection by tests based on DNA polymorphism (Charlesworth et al. 1993), thus raising the possibility that our results could be a byproduct of the distribution of purifying selection across the network. This effect, however, is unlikely to have affected our network-level analyses, given that we combined the results of different positive selection tests and Enard et al. (2014) demonstrated that iHS was insensitive to BGS. Indeed, multivariate analyses confirmed that the relationship between network degree and positive selection was independent of purifying selection (supplementary note, figs. S10 and S11, and tables S7 and S8, Supplementary Material online).

Factors such as gene expression level and breadth (tissue specificity), and the length of the encoded proteins, correlate with both network centralities and the likelihood of detecting positive selection (Anisimova et al. 2002; Lemos et al. 2005; Kim et al. 2007; Kosiol et al. 2008; Alvarez-Ponce 2012; Alvarez-Ponce and Fares 2012) and thus could also represent confounding factors. However, the relationship between network degree and all metrics of positive selection (2Δ*ℓ* and *Z*_F_) and purifying selection (ω and DAF) considered in this study remains unaltered when controlling for these parameters (table 1 and supplementary note and fig. S5, Supplementary Material online).

Our results might also be biased by the incompleteness or low quality of available interactomic data. However, similar results were obtained when a high-quality subnetwork of BioGRID (Stark et al. 2011) or the Human Protein Reference Database (Keshava Prasad et al. 2009) was analyzed (see supplementary note, figs. S6 and S7, and table S5, Supplementary Material online), indicating that our observations are not a byproduct of the quality of network data.

In addition to degree, which is a local measure of network centrality, we used two additional centrality measures that take into account the global position of proteins within the network: Betweenness (the number of shortest paths between other proteins passing through a protein) and closeness (the inverse of the average distance to all other proteins in the network). Similar trends to those observed when using degree were observed in both cases (see supplementary note, figs. S8 and S9, and table S6, Supplementary Material online).

### Assessing the Putative Target of Positive Selection during Recent Human Evolution

**Fig. 3.—:**
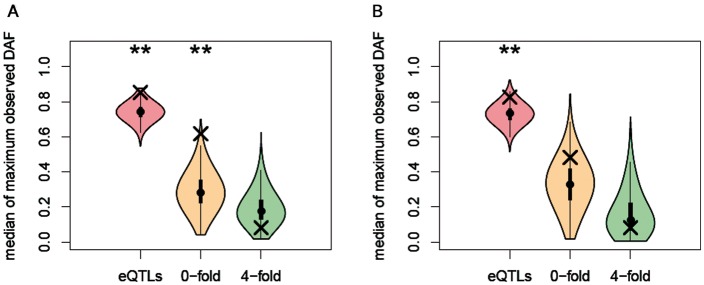
DAF for three sites classes nearby genes with signal of recent positive selection. Crosses represent the median of the maximum DAF observed for three site classes nearby polyPSGs: *cis-*eQTLs, 0-fold degenerated sites and 4-fold degenerated sites. The violin plots represent the distribution of the median of maximum DAF scores observed for a given site class in 10,000 random sets of PIN genes. The analysis was restricted to PIN genes for which the DAF could be calculated for at least one SNP for each of the three site classes. (*A*) Using eQTLs reported by the GEUVADIS consortium (Lappalainen et al. 2013) and located within 100 kb from the associated gene. The polyPSGs set contained 29 genes and the permutations were performed on a set of 358 PIN genes. (*B*) Using eQTLs reported by Liang et al. (2013) and located within 100 kb from the associated gene. The polyPSGs set contained 14 genes and the permutations were performed on a set of 198 PIN genes. Significantly higher median DAF in a site class for polyPSGs as compared with the 10,000 permutations is marked with asterisks. **P < 0.01.

In order to contrast whether positive selection had a stronger impact on the regulatory or on the protein-coding regions of PIN genes during recent human evolution, we compared the DAFs observed for three site classes nearby polyPSGs: *cis-*eQTLs, nonsynonymous (0-fold degenerated sites) and synonymous (4-fold degenerated sites) variants (see Materials and Methods). Following the hard sweep model, we assumed that the variant targeted by positive selection and driving the signal detected using polymorphism data must exhibit an important DAF. We assessed for each site class whether the maximum DAF was higher for polyPSGs than expected in an average PIN gene through 10,000 random samplings (fig. 3). Unsurprisingly, we observed that synonymous sites in polyPSGs do not exhibit extreme DAF. On the contrary, *cis*-eQTLs associated with polyPSGs present higher DAF than expected in the two studied data sets (*P < *0.01; fig. 3). Thus, signals of recent positive selection observed nearby polyPSGs are likely to be driven by variants located in their *cis*-regulatory regions. However, positive selection acting on functional variants located in the protein-coding region may also have driven some of the detected signals, as suggested by the high DAF observed at nonsynonymous sites located in polyPSGs (fig. 3).

### Gene Essentiality and Impact of Positive Selection

To explore whether genes putatively under recent positive selection in our data set (i.e., affected by a hard sweep during recent human evolution) have important fitness effects, we classified the genes under study as viable or lethal using information from The Mouse Genome Database (Bult et al. 2008). Lethal genes present a significantly higher degree than viable genes (Mann–Whitney test; *P* < 0.0001; table 2), in agreement with previous results (Jeong and Albert 2000; Fraser et al. 2002; Iyer et al. 2013). This demonstrates that, as expected, the phenotypic effect of a gene is highly associated with its position within the PIN (for a review, see Olson-Manning et al. 2012). We next compared the scores of positive selection on the PIN genes between the two groups (table 2; fig. 3). As expected, lethal genes have significantly lower DAF and ω scores and higher NI scores (Mann–Whitney test, *P < *0.0001; table 2; fig. 3), indicating that they evolve under higher selective constraints. Moreover, they are more likely to be targeted by recent positive selection, as they exhibit significantly higher positive selection scores in the three human populations (Mann–Whitney test; *P = *0.0047 in YRI, *P* = 0.0009 in CEU, and *P = *0.0235 in CHB; table 2; fig. 3). This indicates that recent positive selection targets genes with the highest effects on fitness. However, during mammal evolution, positive selection is more likely to act on viable genes: 2Δ*ℓ *scores are significantly higher for viable than for lethal genes (Mann–Whitney test; *P < *0.0001; table 2; fig. 3). Similar results were obtained when using the “functional indispensability” score attributed to a specific gene according to its functional and evolutionary properties (Khurana et al. 2013) (table 2; fig. 3).

**Table 2:** Association between Gene Essentiality and Degree and the Impact of Natural Selection

	Lethal versus Viable Genes^a^			Indispensability Score^b^	
	Mean Lethal	Mean Viable	*P* Value	*ρ*	*P *Value
Degree	14.55	7.048	6.62 × 10^−52^***	0.2311	3.03 × 10^−107^***
Positive selection in YRI^c^	6.419	6.154	0.0047**	0.0473	4.34 × 10^−05^***
Positive selection in CEU^c^	6.754	6.350	0.0009***	0.0695	2.00 × 10^−09^***
Positive selection in CHB^c^	6.712	6.423	0.0235*	0.0380	0.0010**
Positive selection in mammals^d^	1.830	2.270	2.03 × 10^−08^***	−0.1157	3.62 × 10^−25^***
Purifying selection in recent humans^e^	0.1041	0.1109	4.66 × 10^−08^***	−0.1131	5.14 × 10^−25^***
Purifying selection in humans^f^	11.81	6.848	2.37 × 10^−09^***	0.1932	3.70 × 10^−29^***
Purifying selection in mammals^g^	0.0768	0.1160	3.70 × 10^−29^***	−0.2600	6.67 × 10^−89^***

^a^Mann–Whitney test to compare the degree or the natural selection score between genes that are essential and genes that are not essential, that is, lethal and viable when knocked out in mice, respectively (data from the Mouse Genome Database [Bult et al. 2008] “MRK_Ensembl_Pheno.rpt” file downloaded on October 7, 2010).

^b^Spearman’s correlation analysis to test for the relationship between degree or the natural selection score and the functional indispensability score (Khurana et al. 2013).

^c,d^High *Z*_F _and 2Δ*ℓ *scores indicate a higher probability of having evolved under positive selection during human and mammal evolution, respectively.

^e^Low DAF scores indicate higher selective constraints during recent human evolution.

^f^High NI scores indicate higher selective constraints during the human lineage evolution.

^g^Low ω scores indicate higher selective constraints during mammal evolution.

*P < 0.05; **P < 0.01; ***P < 0.001.

## Discussion

**Fig. 4.—:**
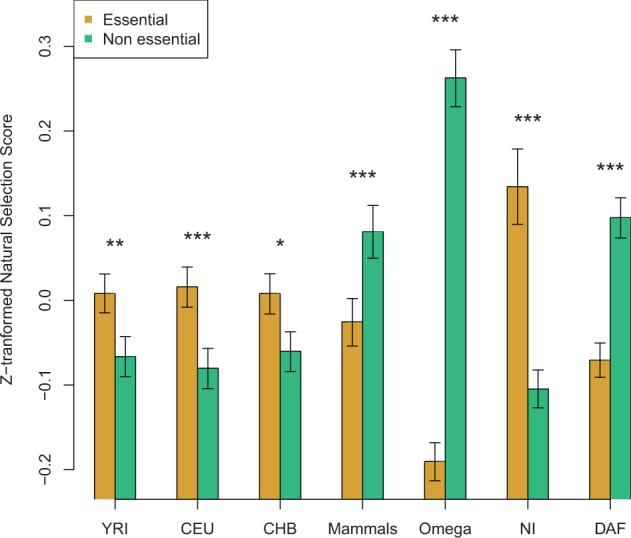
Comparison of the impact of natural selection between essential and nonessential genes. We performed a Mann–Whitney test to compare the selection scores between genes that are lethal (essential, in yellow) and viable (nonessential, in green) when knocked out in mice (data from the Mouse Genome Database [Bult et al. 2008]; “MRK_Ensembl_Pheno.rpt” file downloaded on October 7, 2010). *Z*_F_ and 2Δ*ℓ *scores were used to estimate the likelihood of positive selection in human populations and in mammals, respectively*.* DAF, NI, and ω were used to estimate the impact of purifying selection in recent human populations, in the human lineage, and in mammals, respectively. Lower DAF and ω indicate higher evolutionary constraint estimated from polymorphism and divergence data, respectively, whereas high NI scores indicate higher evolutionary constraint estimated from both polymorphism and divergence data. In order to put all the scores within the same scale, the mean standardized scores are plotted (standardized scores were calculated by subtracting the mean and dividing by the standard deviation). Significant differences between lethal and viable genes pairs are marked with asterisks. *P < 0.05; **P < 0.01; ***P < 0.001.

The results presented here indicate that signatures of positive selection identified following two different methodological frameworks concentrate on different parts of the human PIN: When interrogating mammal divergence data, we observe that positive selection had a greater impact on genes with a lower network centrality, whereas recent, human-specific positive selection (as inferred from polymorphism data) has targeted preferentially genes occupying more central positions in the network. These patterns are independent of several potentially confounding factors (fig. 4).

The signatures of adaptation detected in this study through either a comparative genomics or population genetics approach might correspond to different kinds of changes at the sequence level, a problem with no obvious solution. The maximum-likelihood test used to detect positive selection using divergence data is powerful only in situations in which the gene has experienced recurrent selection events at the coding sequence; adaptation at regulatory sites, however, cannot be detected using this method. Therefore, positive selection during mammal evolution, as inferred here, should be viewed as sequence adaptations that alter the function of proteins recurrently across the mammalian phylogeny. Indeed, the M7 versus M8 test (Nielsen and Yang 1998) will detect an excess, in number, of nonsynonymous substitutions among species; the signal is therefore driven by recurring directional selection. As suggested before (Kim et al. 2007), the interactome periphery may functionally correspond to the cellular periphery (Mi et al. 2013). Indeed, our Gene Ontology enrichment analysis demonstrated that the extracellular region is enriched in proteins encoded by genes showing signals of positive selection in mammals (*P* = 0.0164 after FDR multiple testing correction; supplementary table S9, Supplementary Material online). Gene products acting at the cell periphery are likely to be more exposed to pathogens than genes within the cell, making more likely Red Queen dynamics (Liow et al. 2011) to affect the evolution of peripheral genes.

On the other hand, signatures of selection detected in a genomic region using resequencing data can correspond to unique selective sweeps (not necessarily recurrent) that occurred recently, either at the studied region or at a linked one (e.g., promoters and other regulatory regions). Thus, the putative signals of recent positive selection can be the result of variants that alter protein sequence, but are perhaps more likely to correspond to *cis-*regulatory variants, whose role in recent human evolution seems to have been pivotal (Enard et al. 2014). Moreover, when studying genetic diversity in coding sequences, Hernandez et al. (2011) showed that hard selective sweeps were rare in the human lineage. As protein-coding genes are particularly constrained at the core of the interactome, their regulatory regions may provide the necessary pool of variation for adaptation. In agreement with this hypothesis, we showed that recent positive selection seems to have particularly targeted *cis-*regulatory regions (fig. 3). However, hard sweeps are not the only way for short-term adaptation and soft sweeps, partial sweep and polygenic adaptation are expected to also play a crucial role (for a review, see Pritchard et al. 2010). Such selective events occur through a subtle shift in allele frequency and, thus, are difficult to detect. Standing variants are good candidates for local adaptation that are likely to affect phenotypes in a polygenic manner, having relatively low size effect. We found no significant association between network topology and signals of local adaptation discovered by looking at the correlations between population-specific allele frequencies and environmental variables across the globe (BayENV; Hancock et al. 2011; supplementary notes, Supplementary Material online). Therefore, it seems that local adaptation events through subtle shifts in allele frequency are uniformly distributed across the PIN. Fraser (2013), using BayENV, demonstrated that positive selection events driving subtle shifts in allele frequency were also more likely to occur in *cis-*regulatory regions than in protein-coding genes. Altogether, recent positive selection events detected using polymorphism data are more likely to correspond to adaptation through changes in expression patterns (gene expression level or regulation), whereas selective events detected through divergence analysis may mostly correspond to changes in protein function.

Another line of explanation for the higher impact of recent positive selection in highly connected genes could be that the relaxation of purifying selection in human populations—due to their reduced effective population size (Hughes and Friedman 2010; Subramanian 2013)—may have allowed the spread of some deleterious mutations in genes encoding highly connected proteins. In order to maintain the viability of the organism, compensatory mutations in these genes or in any gene encoding directly interacting partners would have been adaptive (Charlesworth and Eyre-Walker 2007). However, although purifying selection is likely to have been relaxed in recent human evolution, we demonstrated that it remains stronger in highly connected genes (fig. 2*E* and *F*).

The higher centrality of essential genes suggests that the centre of the network may roughly correspond to the most important, influential, and pleiotropic genes of the system. Certain evolutionary mechanisms may promote a higher adaptability at the centre of the network, where the effects of genes on fitness are important, whereas others may promote a higher incidence of positive selection at the periphery. On the one hand, in the 1930s, Ronald Fisher formulated the hypothesis that mutations with large effects on phenotype, such as those with highly pleiotropic effects, should often be deleterious (Fisher 1930; Orr 2005). In agreement with this hypothesis, purifying selection is stronger on genes acting at the centre of molecular networks (Fraser et al. 2002; Hahn and Kern 2005; Vitkup et al. 2006; Alvarez-Ponce 2012; Alvarez-Ponce and Fares 2012) (but see Jordan et al. 2003; Hahn and Kern 2005), a pattern that we have confirmed analyzing both divergence and polymorphism data. As purifying selection quickly removes a high fraction of new mutations at these genes, one would expect positive selection to rarely act on them because of their reduced variability (Olson-Manning et al. 2012). Therefore, we may expect positive selection to target more frequently the periphery of the network. On the other hand, the action of positive selection at genes occupying the centre of the network is not to be discarded. Indeed, signatures of positive selection are frequent at genes occupying relatively important positions in a number of metabolic and signal transduction pathways (Flowers et al. 2007; Dall’Olio et al. 2012; Luisi et al. 2012; Olson-Manning et al. 2013).

Simulation analyses of hypothetical metabolic pathways have shown that, when pathways are far from the fitness optimum, positive selection first targets enzymes lying at the upstream part, and at the branch points of the pathway, which exert greater control over metabolic flux. In turn, when the system approaches its optimum, positive selection tends to concentrate on enzymes with less flux control, and purifying selection constrains the evolution of upstream and branch-point enzymes (Wright and Rausher 2010; Rausher 2012). These observations match the expected pattern of diminishing returns, first proposed by Ronald Fisher in his Geometric Model of Adaptation (Fisher 1930) (FGM), which states that selection tends to act progressively more often on mutations with smaller phenotypic effects as populations approach a peak in the adaptive landscape. A mutation’s effect is measured as a function of both its effect on a given trait and the numbers of phenotypes that are jointly modified by the mutation (pleiotropic effect) (Fisher 1930; Orr 2005), and theoretical models are currently being developed in order to relate the FGM to information on PINs (e.g., see Martin 2014). According to the FGM, events of selection are more likely to be observed on mutations with small phenotypic effects (following a geometric distribution), whereas positive selection on mutations with large effects is most likely to occur during the first steps of adaptation.

The results described in this study can be understood according to both the FGM and the different kinds of advantageous changes detected at the sequence level. Indeed, when focusing at large evolutionary time-scale, that is, during mammal evolution, we are studying the whole process of adaptation acting exclusively on protein-coding genes that made the species fit. Therefore, according to the geometric distribution of the probability of a mutation to be favorable, it is more likely to detect events of adaptation acting on genes with lower effect on fitness, that is, genes encoding proteins with less interacting partners. On the other hand, when focusing at much shorter evolutionary time-scale, that is, during recent human evolution, we are studying the recent adaptation of human populations to a wide range of new environments (e.g., the Mesolithic–Neolithic transition, the human diaspora across the world, etc.). We speculate here that events of strong recent positive selection, as inferred from polymorphism data assuming the hard sweep model, mainly targeted *cis-*regulatory regions of genes with important effects on fitness in order to efficiently tune some specific phenotypes.

In summary, even though the interactome is a raw simplification of the processes that take place within the cell, it contains valuable information on the relative role of the many gene products that interact to sustain life. The position occupied by a protein within an interaction network provides useful information—albeit incomplete—on the phenotypic effects of mutations arising at the encoding gene. Interestingly, we have shown that using this information can also help to better understand the impact of positive selection acting on protein-coding genes and their *cis*-regulatory region. Although network centrality used alone remains a modest predictor of the impact of positive selection, it could be included in an integrative biology approach to shed light on adaptive processes acting on the genome. This study also underscores the fact that the relationship between positive selection and network position is more complex than previously recognized, when positive selection was suggested to mostly act at the network periphery. Indeed, the discovery of the rules governing network evolution may shed light on the dynamics of the evolutionary processes driven by selection. Notably, the distribution of selective events in a large-scale PIN described in this study, which relies on extensive sequence data, can be understood in the light of the Fisher’s Geometric Model of Adaptation. Particularly, results presented here show that the raw material for innovation is also to be found in genes, or in their *cis*-regulatory region, encoding proteins with high network centrality, meaning that they have more pleiotropic effects, are more indispensable and in general are at the basis of strong changes as a result of mutations.

## Supplementary Material

Supplementary data files S1–S4, notes, tables S1–S10, figures S1–S11 are available at *Genome Biology and Evolution *online (http://www.gbe.oxfordjournals.org/).

Supplementary Data

## References

[evv055-B1] 1000 Genomes Project Consortium (2012). An integrated map of genetic variation from 1,092 human genomes. Nature.

[evv055-B2] Agrafioti I (2005). Comparative analysis of the *Saccharomyces cerevisiae* and *Caenorhabditis elegans* protein interaction networks. BMC Evol Biol..

[evv055-B3] Alvarez-Ponce D (2012). The relationship between the hierarchical position of proteins in the human signal transduction network and their rate of evolution. BMC Evol Biol..

[evv055-B4] Alvarez-Ponce D, Fares MA (2014). Why proteins evolve at different rates: the determinants of proteins’ rates of evolution. Natural selection: methods and applications.

[evv055-B5] Alvarez-Ponce D, Fares MA (2012). Evolutionary rate and duplicability in the *Arabidopsis thaliana* protein–protein interaction network. Genome Biol Evol..

[evv055-B6] Anisimova M, Bielawski JP, Yang Z (2002). Accuracy and power of the likelihood ratio test in detecting adaptive molecular evolution. Mol Biol Evol..

[evv055-B7] Bult CJ, Eppig JT, Kadin JA, Richardson JE, Blake JA (2008). The Mouse Genome Database (MGD): mouse biology and model systems. Nucleic Acids Res..

[evv055-B81] Bustamante CD (2005). Natural selection on protein-coding genes in the human genome. Nature.

[evv055-B8] Charlesworth B, Morgan MT, Charlesworth D (1993). The effect of deleterious mutations on neutral molecular variation. Genetics.

[evv055-B9] Charlesworth J, Eyre-Walker A (2007). The other side of the nearly neutral theory, evidence of slightly advantageous back-mutations. Proc Natl Acad Sci U S A..

[evv055-B10] Chen H, Patterson N, Reich D (2010). Population differentiation as a test for selective sweeps. Genome Res..

[evv055-B11] Codoñer FM, Fares MA (2008). Why should we care about molecular coevolution?. Bioinformatics.

[evv055-B12] Coop G (2009). The role of geography in human adaptation. PLoS Genet..

[evv055-B13] Cork JM, Purugganan MD (2004). The evolution of molecular genetic pathways and networks. Bioessays.

[evv055-B14] Cui Q, Purisima E, Wang E (2009). Protein evolution on a human signaling network. BMC Syst Biol..

[evv055-B15] Dall’Olio GM (2012). Distribution of events of positive selection and population differentiation in a metabolic pathway: the case of asparagine N-glycosylation. BMC Evol Biol..

[evv055-B16] Dixon AL (2007). A genome-wide association study of global gene expression. Nat Genet..

[evv055-B17] Do CB, Mahabhashyam MSP, Brudno M, Batzoglou S (2005). ProbCons: Probabilistic consistency-based multiple sequence alignment. Genome Res..

[evv055-B18] Enard D, Messer PW, Petrov DA (2014). Genome-wide signals of positive selection in human evolution. Genome Res..

[evv055-B19] Fay JC, Wu CI (2000). Hitchhiking under positive Darwinian selection. Genetics.

[evv055-B20] Fisher RA (1930). The genetical theory of natural selection.

[evv055-B21] Flicek P (2010). Ensembl’s 10th year. Nucleic Acids Res..

[evv055-B22] Flowers JM (2007). Adaptive evolution of metabolic pathways in *Drosophila*. Mol Biol Evol..

[evv055-B23] Fraser HB (2013). Gene expression drives local adaptation in humans. Genome Res..

[evv055-B24] Fraser HB, Hirsh AE, Steinmetz LM, Scharfe C, Feldman MW (2002). Evolutionary rate in the protein interaction network. Science.

[evv055-B25] Grossman SR (2010). A composite of multiple signals distinguishes causal variants in regions of positive selection. Science.

[evv055-B26] Grossman SR (2013). Identifying recent adaptations in large-scale genomic data. Cell.

[evv055-B27] Hahn MW, Conant GC, Wagner A (2004). Molecular evolution in large genetic networks: does connectivity equal constraint?. J Mol Evol..

[evv055-B28] Hahn MW, Kern AD (2005). Comparative genomics of centrality and essentiality in three eukaryotic protein-interaction networks. Mol Biol Evol..

[evv055-B29] Hall TA (1999). BioEdit: a user-friendly biological sequence alignment editor and analysis program for Windows 95/98/NT. Nucleric Acids Symp Ser..

[evv055-B30] Hancock AM (2011). Adaptations to climate-mediated selective pressures in humans. PLoS Genet..

[evv055-B31] Hernandez RD (2011). Classic selective sweeps were rare in recent human evolution. Science.

[evv055-B32] Hughes AL, Friedman R (2010). More radical amino acid replacements in primates than in rodents: support for the evolutionary role of effective population size. Gene.

[evv055-B33] Iyer S, Killingback T, Sundaram B, Wang Z (2013). Attack robustness and centrality of complex networks. PLoS One.

[evv055-B34] Jeong H, Albert R (2000). The large-scale organization of metabolic networks. Nature.

[evv055-B35] Jordan IK, Wolf Y, Koonin E (2003). No simple dependence between protein evolution rate and the number of protein-protein interactions: only the most prolific interactors tend to evolve slowly. BMC Evol Biol..

[evv055-B36] Karolchik D, Hinrichs AS, Kent WJ (2009). The UCSC Genome Browser.

[evv055-B37] Kelley JL, Madeoy J, Calhoun JC, Swanson W, Akey JM (2006). Genomic signatures of positive selection in humans and the limits of outlier approaches. Genome Res..

[evv055-B38] Kersey PJ (2012). Ensembl Genomes: an integrative resource for genome-scale data from non-vertebrate species. Nucleic Acids Res..

[evv055-B39] Keshava Prasad TS (2009). Human Protein Reference Database—2009 update. Nucleic Acids Res..

[evv055-B40] Khurana E, Fu Y, Chen J, Gerstein M (2013). Interpretation of genomic variants using a unified biological network approach. PLoS Comput Biol..

[evv055-B41] Kim PM, Korbel JO, Gerstein MB (2007). Positive selection at the protein network periphery: evaluation in terms of structural constraints and cellular context. Proc Natl Acad Sci U S A..

[evv055-B42] Kosiol C (2008). Patterns of positive selection in six Mammalian genomes. PLoS Genet..

[evv055-B43] Lappalainen T (2013). Transcriptome and genome sequencing uncovers functional variation in humans. Nature.

[evv055-B44] Lemos B, Bettencourt BR, Meiklejohn CD, Hartl DL (2005). Evolution of proteins and gene expression levels are coupled in *Drosophila* and are independently associated with mRNA abundance, protein length, and number of protein-protein interactions. Mol Biol Evol..

[evv055-B45] Liang L (2013). A cross-platform analysis of 14,177 expression quantitative trait loci derived from lymphoblastoid cell lines. Genome Res..

[evv055-B46] Liao B, Zhang J (2008). Null mutations in human and mouse orthologs frequently result in different phenotypes. Proc Natl Acad Sci U S A..

[evv055-B47] Liow LH, Van Valen L, Stenseth NC (2011). Red Queen: from populations to taxa and communities. Trends Ecol Evol..

[evv055-B48] Lovell SC, Robertson DL (2010). An integrated view of molecular coevolution in protein-protein interactions. Mol Biol Evol..

[evv055-B49] Luisi P (2012). Network-level and population genetics analysis of the insulin/TOR signal transduction pathway across human populations. Mol Biol Evol..

[evv055-B50] MacArthur DG (2012). A systematic survey of loss-of-function variants in human protein-coding genes. Science.

[evv055-B51] Martin G (2014). Fisher’s geometrical model emerges as a property of complex integrated phenotypic networks. Genetics.

[evv055-B52] McDonald JH, Kreitman M (1991). Adaptive protein evolution at the Adh locus in *Drosophila*. Nature.

[evv055-B53] Mi H, Muruganujan A, Thomas PD (2013). PANTHER in 2013: modeling the evolution of gene function, and other gene attributes, in the context of phylogenetic trees. Nucleic Acids Res..

[evv055-B54] Montanucci L, Laayouni H, Bertranpetit J, Fares MA (2014). The network framework of molecular evolution. Natural selection: methods and applications.

[evv055-B55] Morar N, Cookson WOCM, Harper JI, Moffatt MF (2007). Filaggrin mutations in children with severe atopic dermatitis. J Invest Dermatol..

[evv055-B56] Nielsen R, Yang Z (1998). Likelihood models for detecting positively selected amino acid sites and applications to the HIV-1 envelope gene. Genetics.

[evv055-B57] Olson-Manning CF, Lee C-R, Rausher MD, Mitchell-Olds T (2013). Evolution of flux control in the glucosinolate pathway in *Arabidopsis thaliana*. Mol Biol Evol..

[evv055-B58] Olson-Manning CF, Wagner MR, Mitchell-Olds T (2012). Adaptive evolution: evaluating empirical support for theoretical predictions. Nat Rev Genet..

[evv055-B59] Orr HA (2005). The genetic theory of adaptation: a brief history. Nat Rev Genet..

[evv055-B60] Pérez-Bercoff Å, Hudson CM, Conant GC (2013). A conserved mammalian protein interaction network. PLoS One.

[evv055-B61] Pickrell JK (2010). Understanding mechanisms underlying human gene expression variation with RNA sequencing. Nature.

[evv055-B62] Pritchard JK, Pickrell JK, Coop G (2010). The genetics of human adaptation: hard sweeps, soft sweeps, and polygenic adaptation. Curr Biol..

[evv055-B63] Pybus M (2014). 1000 Genomes Selection Browser 1.0: a genome browser dedicated to signatures of natural selection in modern humans. Nucleic Acids Res..

[evv055-B64] Rausher MD (2012). The evolution of genes in branched metabolic pathways. Evolution.

[evv055-B65] Sawyer SA, Hartl DL (1992). Population genetics of polymorphism and divergence. Genetics.

[evv055-B66] Scheinfeldt LB (2009). Population genomic analysis of ALMS1 in humans reveals a surprisingly complex evolutionary history. Mol Biol Evol..

[evv055-B67] Smith JM, Smith NH (1996). Synonymous nucleotide divergence: what is “saturation”?. Genetics.

[evv055-B68] Stark C (2011). The BioGRID Interaction Database: 2011 update. Nucleic Acids Res..

[evv055-B69] Subramanian S (2013). Significance of population size on the fixation of nonsynonymous mutations in genes under varying levels of selection pressure. Genetics.

[evv055-B70] Tajima F (1989). Statistical method for testing the neutral mutation hypothesis by DNA polymorphism. Genetics.

[evv055-B71] Talavera G, Castresana J (2007). Improvement of phylogenies after removing divergent and ambiguously aligned blocks from protein sequence alignments. Syst Biol..

[evv055-B72] Teshima KM, Coop G, Przeworski M (2006). How reliable are empirical genomic scans for selective sweeps?. Genome Res..

[evv055-B73] Vitkup D, Kharchenko P, Wagner A (2006). Influence of metabolic network structure and function on enzyme evolution. Genome Biol..

[evv055-B74] Voight BF, Kudaravalli S, Wen X, Pritchard JK (2006). A map of recent positive selection in the human genome. PLoS Biol..

[evv055-B75] Wagner A (2012). Metabolic networks and their evolution. Adv Med Biol..

[evv055-B76] Wright KM, Rausher MD (2010). The evolution of control and distribution of adaptive mutations in a metabolic pathway. Genetics.

[evv055-B77] Yang Z, Wong WSW, Nielsen R (2005). Bayes empirical Bayes inference of amino acid sites under positive selection. Mol Biol Evol..

[evv055-B78] Zeng K, Shi S, Wu C-I (2007). Compound tests for the detection of hitchhiking under positive selection. Mol Biol Evol..

[evv055-B79] Zhai W, Nielsen R, Slatkin M (2009). An investigation of the statistical power of neutrality tests based on comparative and population genetic data. Mol Biol Evol..

[evv055-B80] Zhang J, He X (2005). Significant impact of protein dispensability on the instantaneous rate of protein evolution. Mol Biol Evol..

