# Supplementary material for: Recent Positive Selection Has Acted on Genes Encoding Proteins with More Interactions within the Whole Human Interactome
Source: Genome Biol Evol. 2015 Apr 2;7(4):1141–54. doi: 10.1093/gbe/evv055 (PMC4419801; doi:10.1093/gbe/evv055)
Supplement: Supplementary Data [file supp_7_4_1141__index.html]

Recent Positive Selection Has Acted On Genes Encoding Proteins With More Interactions Within the Whole Human Interactome — Recent Positive Selection Has Acted on Genes Encoding Proteins with More Interactions within the Whole Human Interactome — Supplementary Data 

# Recent Positive Selection Has Acted on Genes Encoding Proteins with More Interactions within the Whole Human Interactome

## Supplementary Data

files

**Files in this Data Supplement:**

- Supplementary Data - pdf file
