# Supplementary material for: Recent Positive Selection Has Acted on Genes Encoding Proteins with More Interactions within the Whole Human Interactome
Source: Genome Biol Evol. 2015 Apr 2;7(4):1141–54. doi: 10.1093/gbe/evv055 (PMC4419801; doi:10.1093/gbe/evv055)
Supplement: Supplementary Data [file supp_evv055_Luisi-etal_2015-02-18_SuppMat.pdf]

RESEARCH ARTICLE

Supplementary Information

Recent Positive Selection Has Acted On Genes Encoding Proteins With More Interactions Within the Whole Human Interactome

Pierre Luisi[1]†, David Alvarez-Ponce[2,3]†, Marc Pybus[1], Mario A. Fares[2,4], Jaume Bertranpetit[1]* and Hafid Laayouni[1,5]*.

(† These authors contributed equally to this work)

[1]Institute of Evolutionary Biology (Universitat Pompeu Fabra-CSIC), CEXS-UPF-PRBB, Barcelona, Catalonia, Spain.

[2]Integrative Systems Biology Group, Instituto de Biología Molecular y Celular de Plantas, Consejo Superior de Investigaciones Científicas (CSIC)-Universidad Politécnica de Valencia (UPV), Valencia, Spain.

[3]Biology Department, University of Nevada, Reno, NV, USA.

[4]Smurfit Institute of Genetics, University of Dublin, Trinity College, Dublin, Ireland.

[5]Departament de Genètica i de Microbiologia, Grup de Biologia Evolutiva (GBE), Universitat Autonòma de Barcelona, Bellaterra (Barcelona), Spain

* Authors for correspondence.

SUPPLEMENTARY NOTES

Network-Level Analysis Using Different Alignment Sets to Detect Positive Selection from Divergence Data

In order to confirm the relationship observed between the position of proteins in the protein—protein interaction network (PIN) and interspecific positive selection, we used two alternative sets of alignments to which we applied the M7 vs. M8 model (Nielsen & Yang 1998). We first considered the set of 8,697 human genes represented in the PIN with orthologs in 3 to 9 non-human genomes. Alignments were obtained and filtered as described in Material and Methods. We observed that log-likelihood increments ($2\Delta\ell$ scores) from the positive selection test exhibit a significant negative correlation with proteins' degrees (Spearman's rank correlation coefficient, $\rho = -0.0557$; $P < 0.0001$; Supplementary Table 2). In addition, $2\Delta\ell$ values are significantly different in the different groups according to the degree quartiles (non-parametric ANOVA; $P < 0.0001$; Supplementary Table 2; Supplementary Figure 2), and these differences among groups are due to a clear trend towards lower $2\Delta\ell$ scores in groups corresponding to higher degrees (linear trend test on ranks; $P < 0.0001$; Supplementary Table 2; Supplementary Figure 2). Second, we used the alignemnts corresponding to the 5,916 human genes with 1:1 orthologs in all 9 non-human genomes, to which we did not apply any alignment filtering. We also observed a negative correlation between $2\Delta\ell$ and proteins' degrees (Spearman's rank correlation coefficient, $\rho = -0.0772$; $P < 0.0001$; Supplementary Table 2). $2\Delta\ell$ values are also significantly different in the different groups defined according to the degree quartiles (non-parametric ANOVA; $P < 0.0001$; Supplementary Table 2; Supplementary Figure 2), and these differences among groups are due to a clear trend towards lower $2\Delta\ell$ scores in groups corresponding to higher degrees (linear trend test on ranks; $P < 0.0001$; Supplementary Table 2; Supplementary Figure 2). In summary, using these two alternative sets of alignments resulted in qualitatively equivalent results.

Network-Level Analysis Using Different Methods to Detect Positive Selection from Polymorphism Data

In order to confirm the relationship observed between the position of proteins in the PIN and intraspecific positive selection, we broadened the analysis by using separately the three original tests to detect positive selection iHS (Voight et al. 2006); XP-CLR (Chen et al.

2010) and *DH* (Zeng et al. 2007) used to compute the Fisher's combination test score (Z_F). We also used the Composite of Multiple Signals (CMS) method (Grossman et al. 2010) calculated in YRI, CEU and CHB+JPT populations using Pilot1 genotype data from the 1000 Genomes Project (Grossman et al. 2013). For each gene we used the average score (for iHS, XP-CLR and CMS) or the $-\log_{10}(P\text{-value})$ (for *DH*) as summary scores. We then applied a Spearman's correlation analysis between gene degree (number of interactions), as an estimator of network centrality, and these scores. Moreover, genes were classified into four groups delimited by their first, second and third degree quartiles. The median summary scores of the four groups were compared using a non-parametric ANOVA test. We also applied a linear trend test to contrast whether the putative differences among groups were due to a trend towards higher summary scores in groups corresponding to higher degrees (Supplementary Table 3; Supplementary Figure 3).

The Spearman's correlation between the iHS score and degree is positive and significantly different from 0 in the three populations ($\rho \geq 0.0347$; $P \leq 0.0014$; Supplementary Table 3). In addition, iHS values are significantly different in the different groups according to the degree quartiles in all three populations (non-parametric ANOVA; $P \leq 0.0032$; Supplementary Table 3; Supplementary Figure 3), and these differences among groups are due to a clear trend towards higher iHS scores in groups corresponding to higher degrees (linear trend test on ranks; $P \leq 0.0017$; Supplementary Table 3). When using the XP-CLR score, we did not observe such a clear relationship between degree and the impact of positive selection. Indeed, although the Spearman's correlation coefficient is positive in the three populations and significantly different from 0 in YRI and CHB (ρ equal to 0.0385 and 0.0282, respectively; P equal to 0.0007 and 0.0127, respectively; Supplementary Table 3), the non-parametric ANOVA reaches significance only in YRI ($P = 0.0211$), and so does the linear trend test on ranks in YRI and CEU ($P = 0.0028$ and $P = 0.0236$, respectively; Supplementary Table 3; Supplementary Figure 3). Moreover, we also observe an association between degree and the impact of selection as measured by *DH*. Indeed, the Spearman's correlation coefficient is positive and significantly different from 0 in the three populations ($\rho \geq 0.0343$; $P \leq 0.0015$; Supplementary Table 2), and we observed significantly different *DH* values in different degree groups (non-parametric ANOVA; $P \leq 0.0222$; Supplementary Table 3; Supplementary Figure 3), as well as a significant linear trend test on ranks in all three populations ($P \leq 0.0027$; Supplementary Table 3; Supplementary Figure 3). Finally, using the CMS score, the

association appears clearer: we observe significantly positive Spearman's correlation coefficients in all three populations ($\rho \geq 0.0358$; $P \leq 0.0009$; Supplementary Table 3) as well as significant differences in CMS scores among the degree groups due to a clear tendency towards higher CMS values in groups corresponding to higher degrees (non-parametric ANOVA, $P \leq 0.0080$; linear trend test on ranks $P \leq 0.0016$; Supplementary Table 3; Supplementary Figure 3).

In summary, the observed general tendency of central genes to evolve under recent positive selection remains significant when positive selection is inferred separately from different statistics.

Network-Level Analysis for Positive Selection Inferred Using Polymorphism Data in a Subset of Unlinked Genes

To study the impact of positive selection on genes we only used the SNVs located within the genomic region corresponding to the longest transcript. However, it is well known that the regions affected by a selective sweep are large, spanning hundreds of kilobases or even megabases and containing many potential variants driving the signal. Thus, several adjacent genes may be affected by a unique event of selection targeting one particular variant. Therefore, some of the genes showing signals of positive selection in our study may be false positives, even though we do not expect that this bias can affect our network-level analysis, since there is no reason why false positives should tend to concentrate in specific parts of the PIN. To confirm that our study does not suffer from this caveat, we first validated our results using the Composite of Multiple Signals (CMS) method (Grossman et al. 2010) calculated in the YRI, CEU and CHB+JPT populations using the Pilot1 genotype data from the 1000 Genomes Project (Grossman et al. 2010). Although this study used the less accurate Pilot1 data, the implemented method presents the strong advantage of more accurately pinpointing a small number of variants within a large genomic region (Grossman et al. 2010). Thus, using this test we expect to reduce to a great extent the number of falsely detected genes due to genetic hitch-hiking. Our network-level analyses have been confirmed by the use of CMS and, in fact, the association between the impact of selection and network centrality appears to be stronger (see Supplementary Table 3; Supplementary Figure 3; see previous part of this supplementary information).

To further confirm that hitch-hiking does not affect the association between the impact of positive selection and degree, we built a subset of unlinked genes, i.e. not in linkage disequilibrium, for the three populations (YRI, CEU and CHB). For that purpose, in each population, we used the population-specific recombination rates estimated genome-wide (recombination map provided by the 1000 Genomes Project Pilot 1 (The 1000 Genomes Project Consortium 2010)) and defined as a recombination hotspot a region where the observed recombination rates was greater than 10 times the genome average, i.e. greater than 18.36 cM/Mb, 18.55 cM/Mb and 17.61 cM/Mb in YRI, CEU and CHB, respectively. Then, we randomly sampled one PIN gene located between two recombination hotspots. We obtained three subsets of most likely unlinked genes involved in the PIN containing 2792, 3106 and 3107 genes in YRI, CEU and CHB, respectively.

For each gene we used the Z_F score as the likelihood of having been targeted by positive selection in the human populations, and observed that it is significantly positively correlated with degree in all three populations (Spearman's correlation analysis; $\rho \geq 0.0450$; $P \leq 0.0248$; Supplementary Table 4). Moreover, when genes were classified into four groups delimited by the first, second and third degree quartiles, we observed significant differences of summary scores among groups in CEU and CHB (non-parametric ANOVA test, P equal to 0.0036 and 0.0075, respectively; Supplementary Table 4; Supplementary Figure 4). Through a linear trend test on ranks, we concluded that these differences among groups were due to a trend towards higher summary scores in groups corresponding to higher degrees in these two populations ($P \leq 0.0053$; Supplementary Table 4; Supplementary Figure 4). For YRI, although the non-parametric ANOVA was not significant (P = 0.2661), the linear trend test on ranks was marginally significant (P = 0.0482).

Network-Level Analysis Correcting for Putative Confounding Factors

Factors such as gene expression level and breadth (tissues in which a gene is expressed), and the length of the encoded proteins, correlate with both network centralities and the likelihood of detecting natural selection, and hence could potentially have an effect on the observed relationship between the impact of natural selection and the gene centrality in the network (Anisimova et al. 2002; Duret & Mouchiroud 2000; Kim et al. 2007; Kosiol et al. 2008; Pál et al. 2006; Subramanian & Kumar 2004). In order to evaluate the effect of these

factors, we applied a linear regression between the scores used as the likelihood of having been targeted by positive selection during human and mammalian evolution (Z_F and $2\Delta\ell$, respectively), as well as the scores that estimate the strength of purifying selection (DAF, NI and ω) and the mentioned putative confounding factors. The linear regression residuals were then used to perform the correlation analysis in each case. The relationship between positive selection inferred using polymorphism data and degree remains significant in all three populations with a Spearman's correlation coefficient, *ρ,* ranging between 0.0326 (P = 0.0059) in CEU and 0.0451 (P = 0.0001) in YRI (Main text Table 1). Moreover, the non-parametric ANOVA and trend tests on ranks provide similar results when using the linear regression residual instead of the Z_F score, although P-values tend to be higher (Main text Table 1; Supplementary Figure 5). Indeed, the non-parametric ANOVA test is significant in YRI (P = 0.0423) and CEU (P = 0.0240)*,* while it does not reach significance in CHB (P = 0.1006). Moreover, we observe a trend towards higher residuals in groups corresponding to higher degree (Main text Table 1; Supplementary Figure 5). Indeed, in YRI and CHB the linear trend test on ranks reaches significance (P = 0.0053 and P = 0.0239, respectively). Taken together, these observations indicate that the association observed between the Z_F score and degree within the PIN cannot be attributed to the three putative confounding factors. Similarly, the association observed between the impact of positive selection inferred using divergence data (as estimated by $2\Delta\ell$) and degree remains significant when we correct for the three putative confounding factors. Indeed, we observed a significant negative Spearman's partial correlation coefficient (ρ = −0.0340; P = 0.0107; Main text Table 1). Moreover, although the non-parametric ANOVA test is marginally significant (P = 0.0548), the trend test remains significant (P = 0.0122) with lower residuals in groups corresponding to higher degree (Main text Table 1; Supplementary Figure 5). Finally*,* the relationship between purifying selection and degree also remains significant when using the residuals of the linear regression of either DAF or ω with protein length, expression level and expression breadth. Indeed, the correlation tests remain significant (ρ = −0.0668 and −0.1698, respectively; P < 0.0001 in both cases), as well as the non-parametric ANOVA ($P \leq 0.0003$) and the linear trend tests (P < 0.0001). However, the relationship between degree and purifying selection when using the residuals of the linear regression of NI with protein length, expression level and expression breadth, does not remain significant. Indeed, neither the correlation tests (ρ = 0.0314; P = 0.0742), nor the non-parametric ANOVA (P = 0.0713) or the linear trend tests (P

= 0.0852) reach significance (Main text Table 1; Supplementary Figure 5).

Network-Level Analysis Using Different Protein−Protein Interaction Networks

To validate the association between network position and the impact of positive selection, analyses were repeated using two additional high-quality networks: a high-quality (HQ) subnetwork from BioGRID (Stark et al. 2011), in which we retained only interactions discovered by low-throughput techniques, plus those reported in at least two independent high-throughput analyses, and the network from the Human Protein Reference Database (HPRD) (Keshava Prasad et al. 2009), derived from the literature. As in the main analysis, we calculated the number of interactions in which each protein is involved (degree centrality), considering the whole set of non-redundant interactions.

For both the HQ and HPRD networks, the Spearman's correlation coefficient between degree and the recent positive selection Z_F scores is positive and significantly different from 0 in all three populations ($\rho \geq 0.0257$; $P \leq 0.0293$; Supplementary Table 5), except for CHB when using the HPRD data set ($\rho = 0.0229$; P=0.0533). Moreover, for the HQ sub-network the non-parametric ANOVA test is significant in the three populations ($P \leq 0.0095$; Supplementary Table 5; Supplementary Figure 6). These differences among groups are due to a trend towards higher Z_F scores in groups corresponding to higher degree. Indeed, the linear trend test on ranks reaches significance in all three populations ($P \leq 0.0027$; Supplementary Table 5; Supplementary Figure 6). When using the HPRD network, although most of the non-parametric ANOVA tests do not reach significance in YRI and CHB ($P = 0.0401$ in CEU), the overall pattern also points to higher Z_F scores in groups corresponding to higher degrees: the linear trend test on ranks is significant in YRI and CEU (P equal to 0.0364 and 0.0055, respectively; Supplementary Figure 7; Supplementary Table 5) and marginally significant in CHB ($P = 0.0640$).

When studying the association between positive selection as inferred from divergence data (estimated by $2\Delta\ell$) and degree in the HQ and HPRD networks, we observed a negative Spearman's correlation coefficient ($\rho = -0.0620$ and $\rho = -0.0577$, respectively; $P < 0.0001$; Supplementary Table 5). We also observed differences in the $2\Delta\ell$ scores among degree groups (non-parametric ANOVA; $P \leq 0.0011$; Supplementary Table 5; Supplementary Figures 6 and 7), with higher $2\Delta\ell$ scores in groups corresponding to lower degrees. Indeed, the linear trend

test on ranks reaches significance in both networks ($P \leq 0.0001$). Finally, when studying the association between purifying selection and degree in both the HQ and HPRD networks, we observed a significantly negative Spearman's correlation coefficient with DAF and ω ($\rho \leq -0.0488$; $P < 0.0001$; Supplementary Table 5) and a significantly positive Spearman's correlation coefficient with NI ($\rho \geq 0.0714$; $P \leq 0.0002$; Supplementary Table 5). Moreover, we observed clear differences in DAF, NI and ω among degree groups (non-parametric ANOVA, $P \leq 0.0008$), due to a clear tendency towards lower DAF and ω values and higher NI values in groups corresponding to higher degrees (linear trend test on ranks; $P \leq 0.0001$; Supplementary Table 5; Supplementary Figures 6 and 7).

Network-Level Analysis Using Different Centrality Measures

We explored whether the association found between the impact of natural selection and network centrality, as estimated by degree (the number of interactions in which a protein is involved), was also significant when using other centrality measures. For that purpose, we calculated two other centrality measures: betweenness (the number of shortest paths between other proteins passing through a given protein), and closeness (the inverse of the average distance to all other proteins in the network). The association between the impact of natural selection and these network centrality measures remains similar, regardless of the centrality measure considered. Indeed, the Spearman's correlation coefficient between either betweenness or closeness and Z_F, the score used as the likelihood of having been targeted by positive selection in recent human evolution, is significantly positive in all three populations ($\rho \geq 0.0295$; $P \leq 0.0096$; Supplementary Table 6). Moreover, we observed Z_F differences among betweenness groups in the three populations performing a non-parametric ANOVA, which reaches significance in the three populations ($P \leq 0.0332$; Supplementary Table 6; Supplementary Figure 8). These differences are due to a clear tendency for higher Z_F scores in groups corresponding to higher betweenness (linear trend test on ranks, $P \leq 0.0157$; Supplementary Table 6; Supplementary Figure 8). When comparing the Z_F scores among closeness groups, the non-parametric ANOVA test reaches significance in YRI and CHB ($P = 0.0093$ and $P = 0.0116$, respectively; Supplementary Table 6; Supplementary Figure 9). These differences among groups are also due to a trend towards higher Z_F scores in groups corresponding to higher closeness. Indeed, the linear trend test on ranks is significant in all three populations ($P \leq 0.0093$; Supplementary Table 6; Supplementary Figure 9).

When studying the association between positive selection during mammalian evolution (as estimated by $2\Delta\ell$) and network centrality, using both betweenness and closeness, we observed a significantly negative Spearman's correlation coefficient (ρ equal to −0.0645 and −0.0726, respectively; $P < 0.0001$; Supplementary Table 6). We observed differences in the $2\Delta\ell$ scores among betweenness groups (non-parametric ANOVA, $P < 0.0001$; Supplementary Table 6; Supplementary Figure 8), and among closeness groups (non-parametric ANOVA, $P < 0.0001$; Supplementary Table6; Supplementary Figure 9), with a clear tendency for higher $2\Delta\ell$ scores among groups corresponding to lower betweenness or closeness (linear trend test on ranks , $P < 0.0001$; Supplementary Table 6; Supplementary Figures 8-9).

Finally, when studying the association between purifying selection and either betweenness or closeness, we observed significantly negative Spearman's correlation coefficients for DAF and ω ($\rho \leq -0.0641$; $P < 0.0001$; Supplementary Table 6) as well as a significantly positive Spearman's correlation coefficient for NI ($\rho \geq 0.0482$; $P \leq 0.0051$; Supplementary Table 6). Moreover, we observed differences in DAF, NI and ω values among either betweenness or closeness groups (non-parametric ANOVA, $P \leq 0.0221$; Supplementary Table 6; Supplementary Figures 8 and 9), due to a clear tendency towards lower DAF and ω values and higher NI values in groups corresponding to higher centrality measures (linear trend test on ranks; $P \leq 0.0061$; Supplementary Table 6; Supplementary Figures 8 and 9).

Network-Level Analysis for Positive Selection Inferred Using Polymorphism Data Correcting for the Action of Purifying Selection

The action of purifying selection on a genomic region can leave some patterns that are similar to the ones expected under recent positive selection (e.g. an excess of rare variants). Therefore, we wanted to confirm that the association found between positive selection estimated from polymorphism data and degree is not a by-product of the already described association between purifying selection and network centrality. For that purpose, we applied a linear regression between Z_F, the score used as the likelihood of having been targeted by the impact of recent positive selection in human populations, and ω, which estimates the strength of purifying selection during mammalian evolution. The linear regression residuals were then used to perform the analysis. The relationship between positive selection and degree remains

positive in all three populations, with a Spearman's correlation coefficient, ρ, greater than or equal to 0.0195 (Supplementary Table 7). It is significantly different from 0 in YRI and CHB (P equal to 0.0020 and 0.0032, respectively). Moreover, the residuals are marginally different among degree groups (non-parametric ANOVA; P ranging from 0.0371 to 0.0578; Supplementary Table 7; Supplementary Figure 10). These differences are due to a trend towards higher residuals in groups corresponding to higher degrees in YRI and CHB (linear trend test on ranks; P equal to 0.0081 and 0.0100, respectively; Supplementary Table 7; Supplementary Figure 10). In summary, the observed association between Z_F scores and protein degree cannot be attributed to the association between network centrality and the action of purifying selection.

We further confirmed that background selection (BGS), a process that removes neutral variation linked to deleterious mutations, thus reducing levels of polymorphism in regions of high functional density and low recombination (Charlesworth et al. 1993), does not confound the association observed between network centrality and positive selection estimated from polymorphism data. We estimated the level of BGS acting on each gene using two correlates of BGS: GC content and recombination rate. Note that we did not take into account the level of functional constraint because the present study focuses on protein-coding genes. For each gene, we calculated the average of GC content from the 5-mer table downloaded from the UCSC browser (Karolchik et al. 2009) (table "gc5Base" downloaded on the 10th of July, 2013), as well as the average recombination rate from the population-specific recombination rates estimated genome-wide (recombination map provided by the 1000 Genomes Project Pilot 1 (The 1000 Genomes Project Consortium 2010)). We then applied a linear regression between Z_F, the score used as the likelihood of having been targeted by positive selection, and both recombination rate average and GC content average. The linear regression residuals were then used to perform the analysis. The relationship between positive selection and degree remains positive in all three populations, with a Spearman's correlation coefficient, ρ, greater than 0.0369 (Supplementary Table 8) and significantly different from 0 ($P \leq 0.0013$). Moreover, the residuals are different among degree groups in all three populations (non-parametric ANOVA; P ranging from 0.0017 to 0.0089; Supplementary Table 8; Supplementary Figure 11), and these differences are due to a trend towards higher residuals in groups corresponding to higher degree (linear trend test on ranks; P ranging from 0.0007 to 0.0016; Supplementary Table 8; Supplementary Figure 11). Therefore, the association

observed between Z_F scores and degree cannot be attributed to the association between network centrality and the action of BGS.

Network-Level Analysis for Short-Term Positive Selection Through Soft Sweeps

There exist a broad range of methods to detect molecular signals of adaptation, each one taking advantage of specific patterns expected to be observed at a locus that has evolved under positive selection. The methods we used in the present study present the advantage of using only genetic data, and, thus, we could take advantage of the extraordinary SNP density made available by the 1000 Genomes Project (The 1000 Genomes Project Consortium 2012). However, these methods, based on genetic differentiation, linkage disequilibrium and site frequency spectrum, rely on the assumptions that the new beneficial allele is driven in very few generations to high frequencies. Therefore, the signal detected under such assumptions are expected to be the consequence of a selective sweep targeting a mutations with a consequent selective coefficient, most likely due to its important effect size. In order to explore the association between network topology and the impact of positive selection acting through other modes of adaptation (namely soft sweeps, selection of standing variants and polygenic adaptation; for a review see Pritchard et al. 2010), we used the results of a study that scanned the genome for selection signals by identifying the SNPs with the strongest correlations between allele frequencies and climate across 61 worldwide populations (Hancock et al. 2011). This method allows to detect SNPs that went through a similar subtle shift in allele frequency (Hancock et al. 2010) in distant populations living under similar environmental conditions. The positive selection events thus detected are expected to have acted on variants that make smaller contributions to the adaptive trait (Hancock et al. 2010).

We downloaded the results of this study from the DBcline database (http://genapps2.uchicago.edu/dbcline; Hancock et al. 2011) for the absolute latitude and eight environmental variables (see Supplementary Table 6 for the list) and assigned to each gene and each individual environmental variable, the –log10 transformation of the minimum *P*-value reported for the association between the environmental variable and the allele frequencies at SNPs located within the gene. We also computed an extra summary statistic, referred to as "8 Climate Variables" which is the –log10 transformation of the lower *P*-value reported for the association between any of the eight environmental variables and allele frequencies at SNPs located within the gene. We then tested for the correlation between these

local adaptation scores and degree within the PIN. We found no significant correlation (Spearman's correlation test; *P* ranging from 0.0999 to 0.9992 with $-0.0155 < \rho < 0.0195$; Supplementary Table 6). Moreover, we observed six positive correlations and four negative ones. Similar results were obtained when using as a gene-level summary statistic the mean of the score or the proportion of *P*-values below 0.05 (data not shown).

Therefore, there is no evidence for any association between the impact of local adaptation through soft sweep and the network topology, and therefore it seems that local adaptation events through subtle shifts in allele frequencies are uniformly distributed across the PIN.

Supplementary References

Anisimova M, Bielawski JP, Yang Z. 2002. Accuracy and power of the likelihood ratio test in detecting adaptive molecular evolution. Mol. Biol. Evol. 18:1585–1592.

Charlesworth B, Morgan MT, Charlesworth D. 1993. The effect of deleterious mutations on neutral molecular variation. Genetics. 134:1289–303.

Chen H, Patterson N, Reich D. 2010. Population differentiation as a test for selective sweeps. Genome Res. 20:393–402.

Duret L, Mouchiroud D. 2000. Determinants of substitution rates in mammalian genes: expression pattern affects selection intensity but not mutation rate. Mol. Biol. Evol. 17:68–74.

Grossman SR et al. 2010. A Composite of Multiple Signals Distinguishes Causal Variants in Regions of Positive Selection. Science. 327:883–886.

Grossman SR et al. 2013. Identifying recent adaptations in large-scale genomic data. Cell. 152:703–13.

Hancock AM, Alkorta-Aranburu G, Witonsky DB, Di Rienzo A. 2010. Adaptations to new environments in humans: the role of subtle allele frequency shifts. Philos. Trans. R. Soc. Lond. B. Biol. Sci. 365:2459–68.

Hancock AM et al. 2011. Adaptations to Climate-Mediated Selective Pressures in Humans. PLoS Genet. 7:e1001375.

Karolchik D, Hinrichs AS, Kent WJ. 2009. The UCSC Genome Browser. Curr. Protoc. Bioinforma. Chapter1:Unit1:4.

Keshava Prasad TS et al. 2009. Human Protein Reference Database--2009 update. Nucleic Acids Res. 37:D767–72.

Kim PM, Korbel JO, Gerstein MB. 2007. Positive selection at the protein network periphery: evaluation in terms of structural constraints and cellular context. Proc. Natl. Acad. Sci. 104:20274–20279.

Kosiol C et al. 2008. Patterns of positive selection in six Mammalian genomes. PLoS Genet. 4:e1000144.

Mi H, Muruganujan A, Thomas PD. 2013. PANTHER in 2013 : modeling the evolution of gene function , and other gene attributes , in the context of phylogenetic trees. Nucleic Acids Res. 41:377–386.

Nielsen R, Yang Z. 1998. Likelihood models for detecting positively selected amino acid sites and applications to the HIV-1 envelope gene. Genetics. 148:929–936.

Pál C, Papp B, Lercher MJ. 2006. An integrated view of protein evolution. Nat. Rev. Genet. 7:337–48.

Pritchard JK, Pickrell JK, Coop G. 2010. The genetics of human adaptation: hard sweeps, soft sweeps, and polygenic adaptation. Curr. Biol. 20:R208–15.

Stark C et al. 2011. The BioGRID Interaction Database: 2011 update. Nucleic Acids Res. 39:D698–704.

Subramanian S, Kumar S. 2004. Gene expression intensity shapes evolutionary rates of the proteins encoded by the vertebrate genome. Genetics. 168:373–81.

The 1000 Genomes Project Consortium. 2010. A map of human genome variation from population-scale sequencing. Nature. 467:1061–1073.

Voight BF, Kudaravalli S, Wen X, Pritchard JK. 2006. A map of recent positive selection in the human genome. PLoS Biol. 4:e72.

Zeng K, Shi S, Wu C-I. 2007. Compound tests for the detection of hitchhiking under positive selection. Mol. Biol. Evol. 24:1898–908.

Supplementary Table 1. Number of interactions (Degree) for genes with putative signal of positive selection test and for the others.

	Humans[a]				Mammals[c]
	Global[b]	YRI	CEU	CHB	
**Mean degree for genes with signals of positive selection**	9.637	10.34	8.844	9.263	7.578
**Mean degree for genes without signals of positive selection**	8.107	8.438	8.526	8.456	9.122
**Permutation test (*P*-value)[d]**	0.0254*	0.0108*	0.2862	0.0929	0.0067**

[a] Positive selection is invoked when the *P*-value associated to Z_F score is below 5%.
[b] Positive is invoked at global level when the *P*-value associated with the Z_F score is below 5% in at least one of the three studied populations (YRI, CEU or CHB).
[a] Positive selection is invoked when the *P*-value associated to $2\Delta\ell$ score is below 5%.
[d] *P*-values were calculated using permutations. In each permutation a set of genes is randomly drawn, with the sampling size corresponding to the number of genes with signals of positive selection. Then, the average of their degree is compared to the one obtained for the genes with signals of positive selection. *P*- values are computed as the proportion of permutations with an average degree higher or equal to the observed one.
*: $P < 0.05$; **: $P < 0.01$; ***: $P < 0.001$.

Supplementary Table 2: Relationship between degree and the likelihood of having evolved under positive selection during mammal evolution estimated from two alternative alignments sets

		4 to 10 species filtered	10 species unfiltered
**Spearman correlation**[a]	***ρ***	−0.0557	−0.0772
	***P*-value**	2.04×10^{-07}***	2.79×10^{-09}***
**Non-parametric ANOVA**[b]	***F***	9.122	10.11
	***P*-value**	5.04×10^{-06}***	1.22×10^{-06}***
**Trend test on ranks**[b]	***F***	23.05	29.93
	***P*-value**	1.60×10^{-06}***	4.66×10^{-08}***

[a] Spearman correlation between degree and the positive selection score ($2\Delta\ell$) calculated using filtered alignment for 4 to 10 mammal species or unfiltered alignments for 10 species. High scores indicate a higher probability of having evolved under positive selection.

[b] Non-parametric ANOVA and trend tests on ranks performed to contrast whether the medians of the positive selection scores are equal across the degree groups.

*: $P < 0.05$; **: $P < 0.01$; ***: $P < 0.001$.

Supplementary Table 3. Relationship between degree and the likelihood of having evolved under recent positive selection in human populations as estimated from four different statistics.

			YRI	CEU	CHB
iHS	Spearman correlation[a]	*ρ*	0.0347	0.0433	0.0357
		P-value	0.0014**	6.79×10^{-05}***	0.0010**
	Non-parametric ANOVA[b]	*F*	4.609	5.761	4.942
		P-value	0.0032**	0.0006***	0.0020**
	Trend test on ranks[b]	*F*	9.844	15.65	10.41
		P-value	0.0017**	7.70×10^{-05}***	0.0013**
XPCLR	Spearman correlation[a]	*ρ*	0.0385	0.0149	0.0282
		P-value	0.0007***	0.1906	0.0127*
	Non-parametric ANOVA[b]	*F*	3.241	0.7999	1.592
		P-value	0.0211*	0.4941	0.0849
	Trend test on ranks[b]	*F*	8.918	1.999	2.209
		P-value	0.0028**	0.1574	0.0236*
DH	Spearman correlation[a]	*ρ*	0.0343	0.0360	0.0436
		P-value	0.0015**	0.0009***	5.82×10^{-05}***
	Non-parametric ANOVA[b]	*F*	3.206	3.648	4.612
		P-value	0.0222*	0.0121*	0.0032**
	Trend teston ranks[b]	*F*	8.980	9.467	12.91
		P-value	0.0027**	0.0021**	0.0003***
CMS	Spearman correlation[a]	*ρ*	0.0700	0.0566	0.0368
		P-value	1.61×10^{-10}***	2.59×10^{-07}***	0.0009***
	Non-parametric ANOVA[b]	*F*	12.46	8.597	3.945
		P-value	1.09×10^{-09}***	1.07×10^{-05}***	0.0080**
	Trend test on ranks[b]	*F*	37.24	25.61	9.998
		P-value	1.09×10^{-09}***	4.27×10^{-07}***	0.0016**

[a] Spearman correlation between degree and the positive selection score given in the first column.

High scores indicate a higher probability to have evolved under positive selection.

[b] Non-parametric ANOVA and trend tests on ranks performed to contrast whether the medians of the positive selection scores are equal across the degree groups.

*: $P < 0.05$; **: $P < 0.01$; ***: $P < 0.001$.

Supplementary Table 4. Relationship between degree and the likelihood of having evolved under positive selection in human populations using a subset of independent genes.

		YRI	CEU	CHB
**Spearman correlation**[a]	***ρ***	0.0450	0.0600	0.0605
	***P*-value**	0.0248*	0.0017**	0.0014**
**Non-parametric ANOVA**[b]	***F***	1.320	4.521	4.000
	***P*-value**	0.2661	0.0036**	0.0075**
**Trend test on ranks**[b]	***F***	3.908	8.901	7.791
	***P*-value**	0.0482*	0.0029**	0.0053**

We obtained a subset of most likely unlinked genes represented in the network containing 2,792, 3,106 and 3,107 genes in YRI, CEU and CHB, respectively, by randomly sampling one network gene located between two recombination hotspots (defined as a region where the observed recombination rates is greater than 10 times the genome recombination rate average).

[a] Spearman correlation between degree and Z_F in YRI, CEU and CHB.
High Z_F scores indicate a higher probability of having evolved under positive selection.
[b] Non-parametric ANOVA and trend tests on ranks performed to contrast whether the medians of the Z_F score are equal across the degree groups (calculated on the whole set of genes).
*: $P < 0.05$; **: $P < 0.01$.

Supplementary Table 5. Relationship between degree and the impact of natural selection using two alternative high-quality protein−protein interaction networks.

			Positive selection				Purifying selection		
			YRI	CEU	CHB	Mammals	Recent Humans	Humans	Mammals
High-Quality network from BioGRID	Spearman correlation[a]	ρ	0.0379	0.0420	0.0441	−0.0620	−0.0715	0.0731	−0.1730
		P-value	0.0034**	0.0012**	0.0006***	2.53×10^{-05}***	5.82×10^{-09}***	0.0002***	5.50×10^{-32}***
	Non-parametric ANOVA[b]	*F*	3.819	4.208	4.289	5.363	10.29	5.588	45.31
		P-value	0.0095**	0.0056**	0.0050**	0.0011**	9.38×10^{-07}***	0.0008***	7.56×10^{-29}***
	Trend test on ranks[b]	*F*	9.492	9.557	9.039	14.91	28.06	15.21	130.9
		P-value	0.0021**	0.0020**	0.0027**	0.0001***	1.21×10^{-07}***	9.83×10^{-05}***	6.51×10^{-30}***
Network from Human Protein Reference Database	Spearman correlation[a]	ρ	0.0257	0.0364	0.0229	−0.0577	−0.0488	0.0714	−0.1353
		P-value	0.0293*	0.0021**	0.0533	1.04×10^{-05}***	6.88×10^{-06}***	3.68×10^{-05}***	7.97×10^{-21}***
	Non-parametric ANOVA[b]	*F*	2.223	2.769	1.435	5.733	5.663	5.746	27.39
		P-value	0.0833	0.0401*	0.2304	0.0007***	0.0007***	0.0006***	1.49×10^{-17}***
	Trend test on ranks[b]	*F*	4.380	7.721	3.431	16.90	14.85	16.22	80.29
		P-value	0.0364*	0.0055**	0.0640	3.99×10^{-05}***	0.0001***	5.75×10^{-05}***	4.56×10^{-19}***

[a] Spearman correlation between degree and selection scores (Z_F for positive selection in YRI, CEU and CHB populations; $2\Delta\ell$ for positive selection in mammals; DAF for purifying selection during recent human evolution; NI for purifying selection in the human lineage; and ω for purifying selection in mammals). High Z_F and $2\Delta\ell$ scores indicate a higher probability of having evolved under positive selection. Low DAF and ω scores indicate higher selective constraint during human and mammalian evolution, respectively, while high NI scores indicate higher evolutionary constraint estimated from both polymorphism and divergence data.

[b] Non-parametric ANOVA and trend tests on ranks performed to contrast whether the medians of the natural selection scores are equal across the degree groups.

*: *P*-value < 0.05; **: *P*-value < 0.01; ***: *P*-value < 0.001.

Supplementary Table 6. Relationship between network centrality and the impact of natural selection using betweenness and closeness.

			Positive selection				Purifying selection		
			**YRI**	**CEU**	**CHB**	**Mammals**	**Recent Humans**	**Humans**	**Mammals**
**Betweenness**	**Spearman correlation**[a]	***ρ***	0.0295	0.0353	0.0385	−0.0645	−0.0641	0.0562	−0.1848
		***P*-value**	0.0096**	0.0020**	0.0007***	6.84×10^{-07}***	3.15×10^{-09}***	0.0011**	4.92×10^{-46}***
	**Non-parametric ANOVA**[b]	***F***	3.406	4.380	5.677	11.33	15.34	3.818	91.36
		***P*-value**	0.0332*	0.0126*	0.0034**	1.23×10^{-05}***	2.25×10^{-07}***	0.0221*	8.56×10^{-40}***
	**Trend test on ranks**[b]	***F***	5.835	8.728	10.57	22.41	30.65	7.637	182.1
		***P*-value**	0.0157*	0.0031**	0.0012**	2.26×10^{-06}***	3.18×10^{-08}***	0.0058**	7.04×10^{-41}***
**Closeness**	**Spearman correlation**[a]	***ρ***	0.0413	0.0332	0.0427	−0.0726	−0.0802	0.0482	−0.1670
		***P*-value**	0.0003***	0.0037**	0.0002***	2.28×10^{-08}***	1.25×10^{-13}***	0.0051**	3.30×10^{-38}***
	**Non-parametric ANOVA**[b]	***F***	3.839	2.3422	3.678	9.923	16.78	3.976	54.90
		***P*-value**	0.0093**	0.0711	0.0116*	1.60×10^{-06}***	7.29×10^{-11}***	0.0077**	5.46×10^{-35}***
	**Trend test on ranks**[b]	***F***	9.702	6.769	10.54	26.41	49.87	7.530	153.9
		***P*-value**	0.0018**	0.0093**	0.0012**	2.85×10^{-07}***	1.77×10^{-11}***	0.0061**	6.59×10^{-35}***

[a] Spearman correlation between degree and natural selection scores (Z_F for positive selection in the YRI, CEU and CHB populations; $2\Delta\ell$ for positive selection in mammals; DAF for purifying selection during recent human evolution; NI for purifying selection in the human lineage; and ω for purifying selection in mammals). High Z_F and $2\Delta\ell$ scores indicate a higher probability of having evolved under positive selection as inferred from polymorphsim and divergence data, respectively. Low DAF and ω scores indicate higher evolutionary constraint estimated from polymorphism and divergence data, respectively, while high NI scores indicate higher evolutionary constraint estimated from both polymorphism and divergence data.

[b] Non-parametric ANOVA and trend tests on ranks performed to contrast whether the medians of the natural selection scores are equal across the connectivity measure groups. For Betweenness, the 1st and 2nd quartiles were merged due to the uneven distribution of values.

*: *P*-value < 0.05; **: *P*-value < 0.01; ***: *P*-value < 0.001.

Supplementary Table 7. Relationship between degree and the impact of recent positive selection in human populations controlling for ω in mammals.

		YRI	CEU	CHB
**Spearman correlation**[a]	***ρ***	0.0432	0.0195	0.0412
	***P*-value**	0.0020**	0.1655	0.0032**
**Non-parametric ANOVA**[b]	***F***	2.51	2.827	2.499
	***P*-value**	0.0569	0.0371*	0.0578
**Trend test on ranks**[b]	***F***	7.012	2.032	6.646
	***P*-value**	0.0081**	0.1541	0.0100**

In order to test for an association between degree and the impact of positive selection in humans controlling for ω, we used the Z_F as the likelihood of having been targeted by positive selection. We then applied a linear regression between this score and ω. High Z_F values indicate a higher probability of having evolved under positive selection. Low ω scores indicate higher selective constraint. The linear regression residuals were then used to perform the Spearman's correlation analysis, the non-parametric ANOVA and the linear trend test on rank.

[a] Spearman correlation between degree and the residuals.

[b] Non-parametruc ANOVA and trend tests performed to contrast whether the medians of the residuals across the degree groups.

*: $P < 0.05$; **: $P < 0.01$; ***: $P < 0.001$.

Supplementary Table 8. Relationship between degree and the impact of recent positive selection in human populations controlling for covariates of background selection.

		YRI	CEU	CHB
**Spearman correlation[a]**	***ρ***	0.0427	0.0369	0.0428
	***P*-value**	0.0002***	0.0013**	0.0002***
**Non-parametric ANOVA[b]**	***F***	3.872	5.043	4.110
	***P*-value**	0.0089**	0.0017**	0.0064**
**Trend test on ranks[b]**	***F***	11.53	9.947	11.48
	***P*-value**	0.0007***	0.0016**	0.0007***

In order to test for an association between degree and the impact of positive selection in humans controlling for background selection, we used Z_F as the likelihood of having been targeted by positive selection. We then applied a linear regression between this score and both population-specific recombination rate average across the gene and GC content average across the gene. High Z_F values indicate a higher probability of having evolved under positive selection.

The linear regression residuals were then used to perform the Spearman's correlation analysis, the ANOVA and the linear trend test.

[a] Spearman correlation between degree and the residuals.

[b] Non-parametric ANOVA and trend tests performed to contrast whether the medians of the residuals across the degree groups.

*: $P < 0.05$; **: $P < 0.01$; ***: $P < 0.001$.

Supplementary Table 9. Cellular compartment enrichment in signals of positive selection in mammals detected using divergence data.

**Compartment**	**# Genes Tested**[a]	**# Genes Observed**[b]	**# Genes Expected**	**Direction**	***P*-value**	***Q*-value**[c]
Extracellular region	124	25	11.6	+	0.0004	0.0164
Extracellular matrix	72	14	6.74	+	0.0091	0.2039
Unclassified	5088	459	476.09	−	0.0230	0.3450
Plasma membrane	93	14	8.7	+	0.0583	0.5908
MHC protein complex	5	2	0.47	+	0.0806	0.5908
Microvillus	1	1	0.09	+	0.0893	0.5908
Ribonucleoprotein complex	58	2	5.43	−	0.0919	0.5908
Mitochondrion	22	0	2.06	−	0.1270	0.7144
Neuron projection	8	2	0.75	+	0.1730	0.7920
Macromolecular complex	235	18	21.99	−	0.2280	0.7920
Dendrite	3	1	0.28	+	0.2450	0.7920
Membrane	150	17	14.04	+	0.2450	0.7920
Cytosol	14	0	1.31	−	0.2690	0.7920
Mitochondrial inner membrane	14	0	1.31	−	0.2690	0.7920
Cell projection	11	2	1.03	+	0.2750	0.7920
Cell part	629	62	58.86	+	0.3520	0.7920
Cytoskeleton	271	23	25.36	−	0.3630	0.7920
Nucleus	35	4	3.27	+	0.4140	0.7920
Proton-transporting ATP synthase complex	6	1	0.56	+	0.4300	0.7920
Cytoplasm	152	13	14.22	−	0.4390	0.7920
Cell junction	27	3	2.53	+	0.4630	0.7920
Intermediate filament cytoskeleton	17	2	1.59	+	0.4720	0.7920
Intracellular	572	53	53.52	−	0.5060	0.7920
Protein complex	177	16	16.56	−	0.5100	0.7920
Actin cytoskeleton	145	13	13.57	−	0.5100	0.7920
Organelle	389	36	36.4	−	0.5170	0.7920
Ribosome	7	0	0.65	−	0.5190	0.7920
Microtubule	80	7	7.49	−	0.5260	0.7920
Integral to membrane	8	1	0.75	+	0.5270	0.7920
Vesicle coat	17	1	1.59	−	0.5280	0.7920
SNARE complex	14	1	1.31	−	0.6230	0.8536

Chromosome	5	0	0.47	−	0.6260	0.8536
Cytoplasmic membrane-bounded vesicle	5	0	0.47	−	0.6260	0.8536
Synapse	4	0	0.37	−	0.6880	0.8600
Tubulin complex	4	0	0.37	−	0.6880	0.8600
Heterotrimeric G-protein complex	4	0	0.37	−	0.6880	0.8600
Vacuole	3	0	0.28	−	0.7550	0.8882
Apical part of cell	2	0	0.19	−	0.8290	0.8882
Nuclear chromosome	2	0	0.19	−	0.8290	0.8882
Lysosome	2	0	0.19	−	0.8290	0.8882
Cilium	2	0	0.19	−	0.8290	0.8882
Tight junction	2	0	0.19	−	0.8290	0.8882
Endoplasmic reticulum	1	0	0.09	−	0.9110	0.9110
Peroxisome	1	0	0.09	−	0.9110	0.9110
Protein-DNA complex	1	0	0.09	−	0.9110	0.9110

Enrichment analysis performed using the PANTHER statistical over-representation test (Mi et al. 2013) for GO Cellular Compartment for the 554 genes with putative signatures of positive selection in mammals ($P < 0.05$ for the M7 vs. M8 test) and using as a reference set the 5,916 PIN genes for which the $2\Delta\ell$ score could be computed.

[a] Number of genes with signals of positive selection in mammals represented in the GO database.

[b] Number of genes in the reference set represented in the GO database.

[b] FDR *P*-value correction for multiple testing.

Supplementary Table 10. Cellular compartment enrichment in signals of positive selection in recent human evolution detected using polymorphism data.

Compartment	# Genes Tested[a]	# Genes Observed[b]	# Genes Expected	Direction	*P*-value	*Q*-value[c]
Protein complex	246	64	49.28	+	0.0230	0.4389
Macromolecular complex	322	81	64.51	+	0.0238	0.4389
Plasma membrane	132	17	26.45	−	0.0332	0.4389
Extracellular region	177	25	35.46	−	0.0399	0.4389
Vesicle coat	25	9	5.01	+	0.0683	0.6010
Integral to membrane	11	0	2.2	−	0.1100	0.6698
Cell junction	45	5	9.02	−	0.1140	0.6698
Extracellular matrix	122	19	24.44	−	0.1560	0.6698
Microtubule	109	27	21.84	+	0.1570	0.6698
Membrane	202	34	40.47	−	0.1710	0.6698
Actin cytoskeleton	215	39	43.07	−	0.2960	0.6698
Cytoplasm	182	40	36.46	+	0.2980	0.6698
Cytosol	14	4	2.8	+	0.3090	0.6698
Intracellular	748	156	149.86	+	0.3110	0.6698
Nuclear chromosome	6	2	1.2	+	0.3380	0.6698
Mitochondrion	24	6	4.81	+	0.3500	0.6698
Ribonucleoprotein complex	76	17	15.23	+	0.3570	0.6698
Nucleus	43	10	8.61	+	0.3620	0.6698
Organelle	521	108	104.38	+	0.3710	0.6698
Chromosome	11	3	2.2	+	0.3780	0.6698
SNARE complex	16	2	3.21	−	0.3780	0.6698
Cell part	819	168	164.08	+	0.3850	0.6698
Ribosome	7	2	1.4	+	0.4090	0.6698
Proton-transporting ATP synthase complex	7	2	1.4	+	0.4090	0.6698
Synapse	4	0	0.8	−	0.4490	0.6698
Unclassified	6497	1304	1301.63	+	0.4490	0.6698
Neuron projection	9	1	1.8	−	0.4620	0.6698
Cell projection	14	2	2.8	−	0.4680	0.6698
Heterotrimeric G-protein complex	14	2	2.8	−	0.4680	0.6698
Cytoskeleton	390	79	78.13	+	0.4760	0.6698
Mitochondrial inner	18	4	3.61	+	0.4860	0.6698

membrane						
Intermediate filament cytoskeleton	28	6	5.61	+	0.4900	0.6698
Tubulin complex	14	3	2.8	+	0.5320	0.6698
Vacuole	3	0	0.6	−	0.5480	0.6698
Dendrite	3	0	0.6	−	0.5480	0.6698
Cilium	3	0	0.6	−	0.5480	0.6698
Apical part of cell	2	0	0.4	−	0.6700	0.7559
Lysosome	2	0	0.4	−	0.6700	0.7559
Tight junction	2	0	0.4	−	0.6700	0.7559
Cytoplasmic membrane-bounded vesicle	5	1	1	−	0.7350	0.7888
MHC protein complex	5	1	1	−	0.7350	0.7888
Peroxisome	1	0	0.2	−	0.8180	0.8180
Microvillus	1	0	0.2	−	0.8180	0.8180
Protein-DNA complex	1	0	0.2	−	0.8180	0.8180

Enrichment analysis performed using the PANTHER statistical over-representation test (Mi et al. 2013) for GO Cellular Compartment for the 1,521 genes with putative signatures of positive selection in any of the three human populations (*P* < 0.05 for the Fisher's combination test) and using as a reference set the 7,603 PIN genes for which the Z_F score could be computed.

[a] Number of genes with signals of recent positive selection in humans represented in the GO database.

[b] Number of genes in the reference set represented in the GO database.

[b] FDR *P*-value correction for multiple testing.

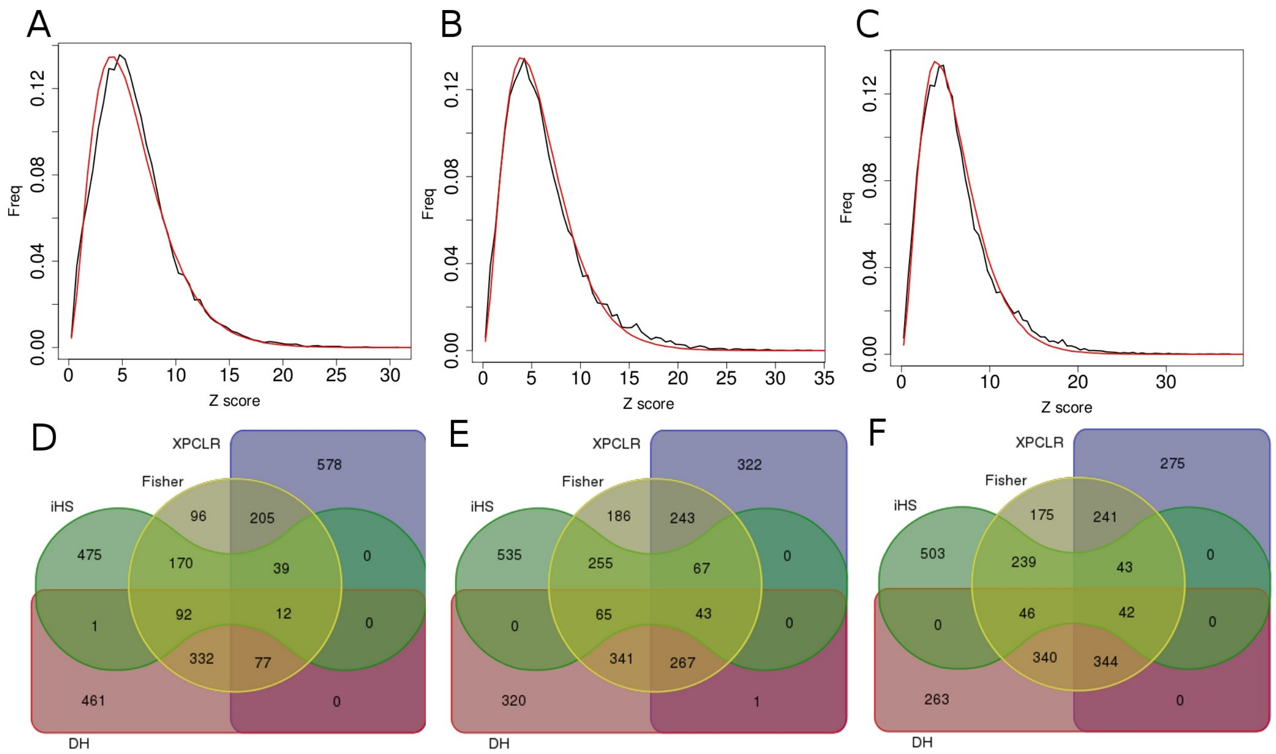

Supplementary Figure 1. Confirmation of the accuracy of the Fisher's combination test score. **A-C**: Comparison of the Fisher's combination Z_F score distribution observed for the genes within the interactome and the genome background set (in black) to the $\chi^2_{(6)}$ expected distribution (in red) in YRI, CEU and CHB populations, respectively. **D-F**: Venn diagram of the genes with a signal of positive selection (*P*-value < 0.05) for the four tests in YRI, CEU and CHB, respectively.

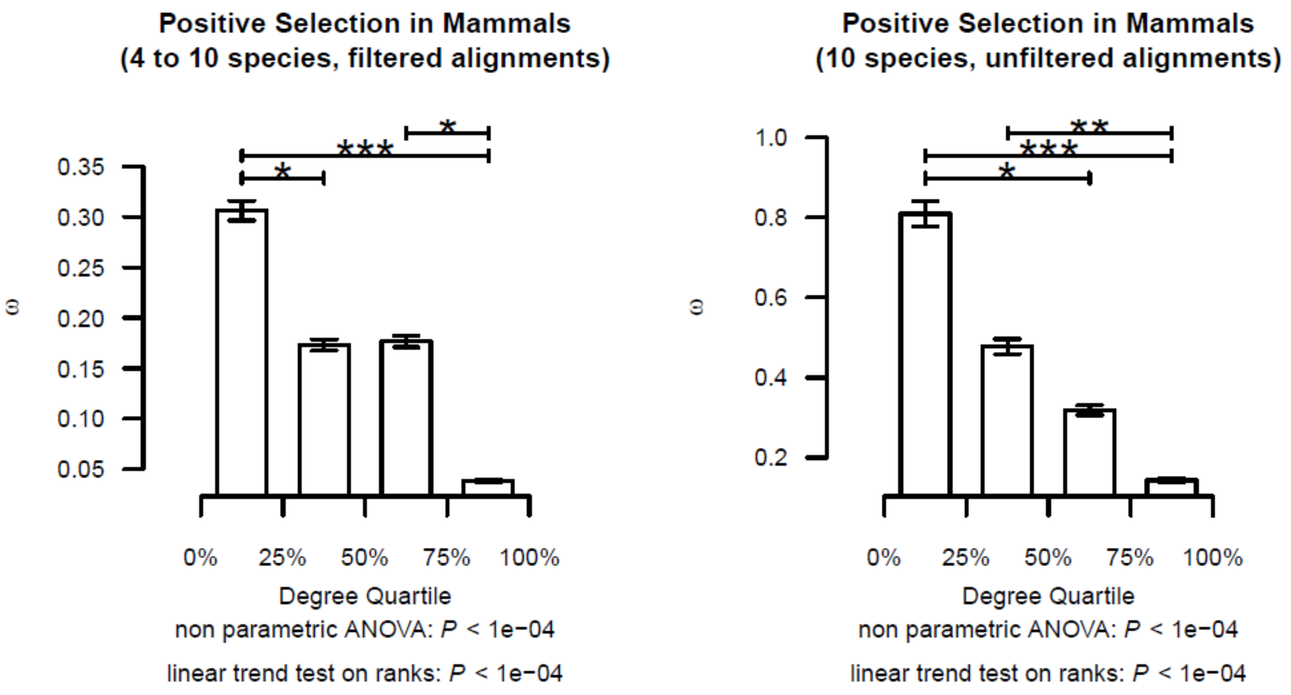

Supplementary Figure 2. Impact of positive selection in mammals as measured on two alternative alignment sets among groups of genes divided according to the degree quartiles. $2\Delta\ell$ scores were used to estimate the impact of positive selection in mammals. Genes were classified into four groups according to the degree quartiles calculated in the network. The median of the positive selection score ± one median absolute deviation within each group is represented in the *y*-axis. A non-parametric ANOVA analysis was performed to contrast whether the medians of the positive selection scores were equal across the groups. A trend test on ranks was also carried out to test for a linear relationship between the four quartiles (encoded from 1 to 4) and natural selection scores. A Tukey's honestly significant difference test was further applied to test for all pairwise differences. Significantly different pairs are marked with asterisks. *: $P < 0.05$; **: $P < 0.01$; ***: $P < 0.001$.

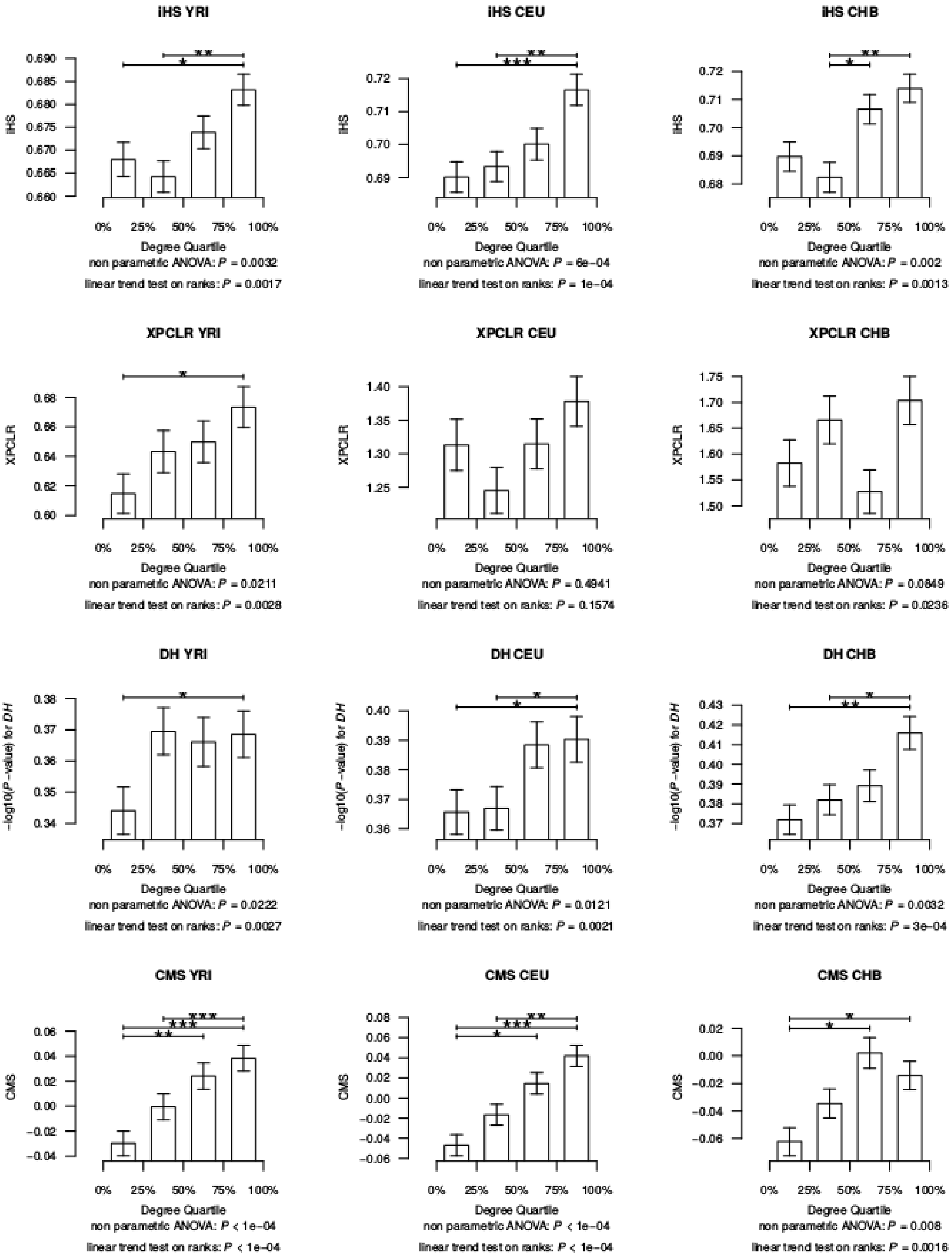

Supplementary Figure 3. Impact of positive selection in human populations as measured by four different tests based on polymorphism data among groups of genes divided according to the degree quartiles.

Genes were classified into four groups according to the degree quartiles calculated in the network. The median of the positive selection score ± one median absolute deviation within each group is

represented in the y-axis. A non-parametric ANOVA analysis was performed to contrast whether the medians of the positive selection scores were equal across the groups. A trend test on ranks was also been carried out to test for a linear relationship between the four quartiles (encoded from 1 to 4) and natural selection scores. A Tukey's honestly significant difference test was further applied to test for all pairwise differences. Significantly different pairs are marked with asterisks. *: $P < 0.05$; **: $P < 0.01$; ***: $P < 0.001$.

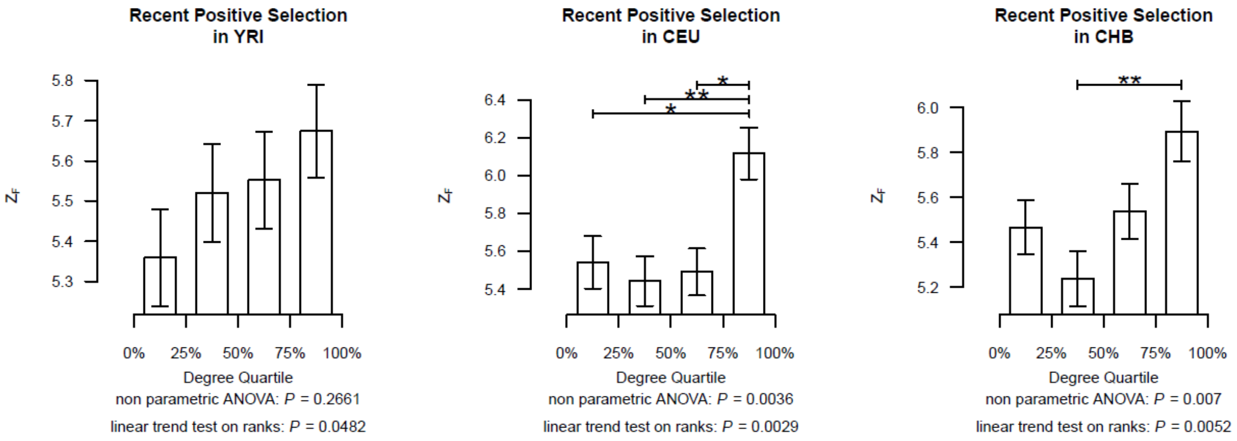

Supplementary Figure 4. Impact of positive selection during recent human evolution among groups of genes classified according to their degree using a subset of independently evolving genes.

We obtained a subset of most likely unlinked genes involved in the network containing 2,793, 3,107 and 3,108 genes in YRI, CEU and CHB, respectively, by randomly sampling one network gene located between two recombination hotspots (defined as a region where the observed recombination rates is greater than 10 times the genome recombination rate average). Genes were classified into four groups according to the degree quartiles. The median of the Z_F scores ± one median absolute deviation within each group is represented in the *y*-axis. A non-parametric ANOVA analysis was performed to contrast whether the median scores are equal across the groups. A trend test on ranks was also carried out to test for a linear relationship between the four groups (encoded from 1 to 4) and natural selection scores. A Tukey's honestly significant difference test was further applied to test for all pairwise differences. Significantly different pairs are marked with asterisks. *: $P < 0.05$; **: $P < 0.01$; ***: $P < 0.001$.

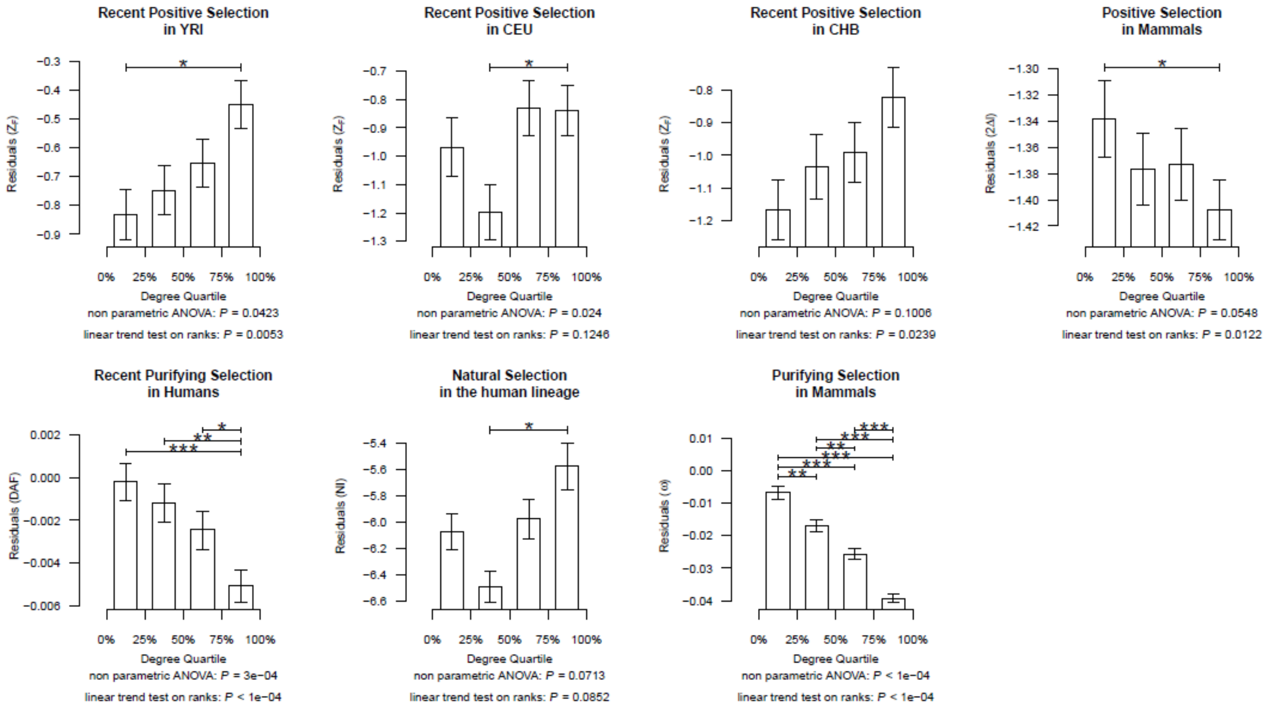

Supplementary Figure 5. Impact of natural selection among groups of genes classified according to their degree controlling for confounding factors.

Z_F and $2\Delta\ell$ were used to estimate the impact of positive selection in human populations and in mammals, respectively. DAF, NI and ω were used to estimate the impact of purifying selection in recent human populations, in the human lineage and in mammals, respectively. Lower DAF and ω values indicate higher evolutionary constraint estimated from polymorphism and divergence data, respectively, while higher NI values indicate higher evolutionary constraint estimated from both polymorphism and divergence data. In order to test for an association between degree and positive selection scores controlling simultaneously for protein length, expression level and expression breadth, we applied a linear regression between positive selection scores and these factors. The linear regression residuals were then used to perform the Spearman's correlation analysis, the non-parametric ANOVA and the linear trend test on ranks. Genes were classified into four groups according to the degree quartiles. The median of the residuals ± one median absolute deviation within each group are represented in the *y*-axis. A non-parametric ANOVA analysis was performed to contrast whether the medians of the scores are equal across the groups. A trend test was carried out to test for a linear relationship between the four groups (encoded from 1 to 4) and natural selection scores. A Tukey's honestly significant difference test was further applied to test for all pairwise differences. Significantly different pairs are marked with asterisks. *: $P < 0.05$; **: $P < 0.01$; ***: $P < 0.001$.

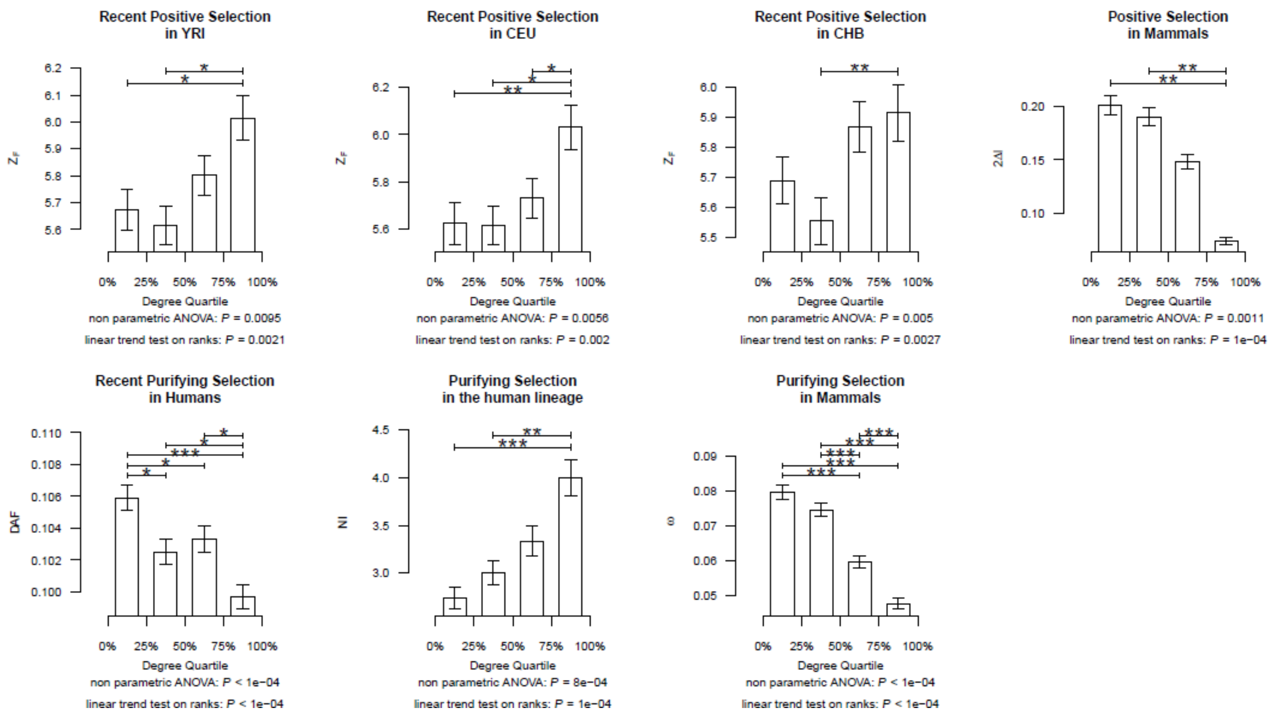

Supplementary Figure 6. Impact of natural selection among groups of genes classified according to their degree in the BioGRID high quality network.

Genes were classified into four groups according to the degree quartiles calculated in the network HQ. The median of the positive selection score used as likelihood of having been targeted by natural selection ± one median absolute deviation within each group is represented across the *y*-axis. Z_F and $2\Delta\ell$ were used to infer the impact of positive selection in human populations and in mammals, respectively. DAF, NI and ω were used to estimate the impact of purifying selection in recent human populations, in the human lineage and in mammals, respectively. Lower DAF and ω values indicate higher evolutionary constraint estimated from polymorphism and divergence data, respectively, while higher NI values indicate higher evolutionary constraint estimated from both polymorphism and divergence data. A non-parametric ANOVA analysis was been performed to contrast whether the medians of the scores were equal across the groups. A trend test on ranks was also carried out to test for a linear relationship between the four groups (encoded from 1 to 4) and natural selection scores. A Tukey's honestly significant difference test was further applied to test for all pairwise differences. Significantly different pairs are marked with asterisks. *: $P < 0.05$; **: $P < 0.01$; ***: $P < 0.001$.

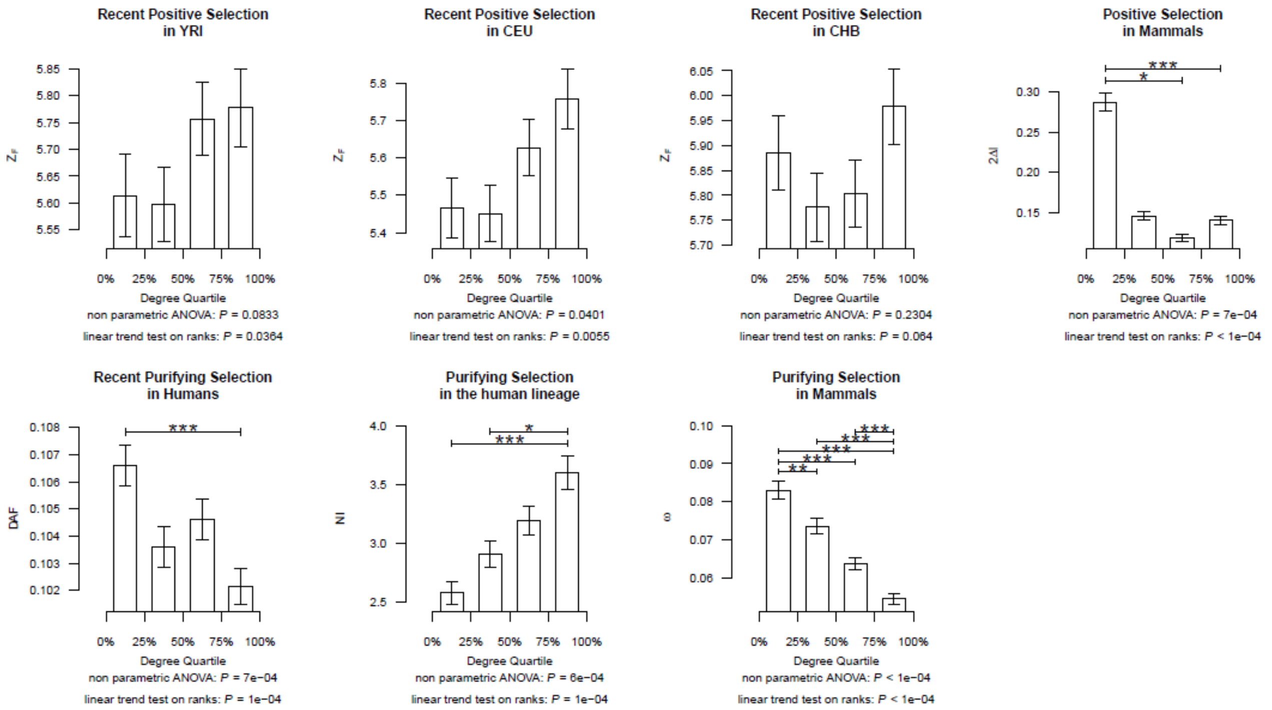

Supplementary Figure 7. Impact of natural selection among groups of genes classified according to their degree in the Human Protein Reference Database network.

Genes were classified into four groups according to the degree quartiles calculated in the HPRD network. The median of the positive selection scores ± one median absolute deviation within each group is represented across the *y*-axis. Z_F and $2\Delta\ell$ were used to estimate the likelihood of having been targeted by positive selection in human populations and in mammals, respectively. DAF, NI and ω were used to estimate the impact of purifying selection in recent human populations, in the human lineage and in mammals, respectively. Lower DAF and ω values indicate higher evolutionary constraint estimated from polymorphism and divergence data, respectively, while higher NI values indicate higher evolutionary constraint estimated from both polymorphism and divergence data. A non-parametric ANOVA analysis was performed to contrast whether the medians of the positive selection scores are equal across the groups. A trend test on ranks was also carried out to test for a linear relationship between the four groups (encoded from 1 to 4) and natural selection scores. A Tukey's honestly significant difference test was further applied to test for all pairwise differences. Significantly different pairs are marked with asterisks. *: $P < 0.05$; **: $P < 0.01$; ***: $P < 0.001$.

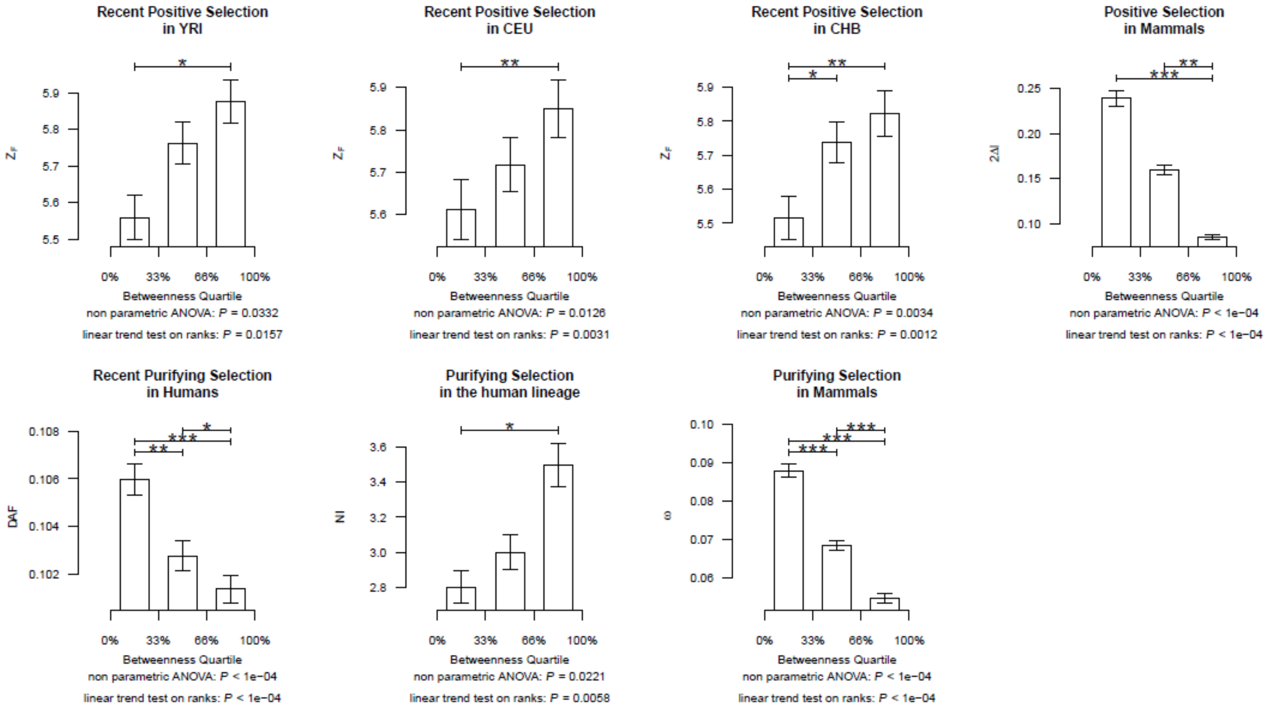

Supplementary Figure 8. Impact of natural selection among groups of genes classified according to their betweenness in the BioGRID network.

Genes were classified into four groups according to the betweenness quartiles calculated in the interactome. The 1st and 2nd groups were merged due to the uneven distribution of values. The median of the positive selection scores ± one median absolute deviation within each group is represented across the *y*-axis. Z_F and $2\Delta\ell$ were used to estimate the likelihood of having been targeted by positive selection in human populations and in mammals, respectively. DAF, NI and ω were used to estimate the impact of purifying selection in recent human populations, in the human lineage and in mammals, respectively. Lower DAF and ω values indicate higher evolutionary constraint estimated from polymorphism and divergence data, respectively, while higher NI values indicate higher evolutionary constraint estimated from both polymorphism and divergence data. A non-parametric ANOVA analysis was performed to contrast whether the medians of the scores are equal across the groups. A trend test on ranks was also carried out to test for a linear relationship between the four quartiles (encoded from 1 to 3) and natural selection scores. A Tukey's honestly significant difference test has been further applied to test for all pairwise differences. Significantly different pairs are marked with asterisks. *: $P < 0.05$; **: $P < 0.01$; ***: $P < 0.001$.

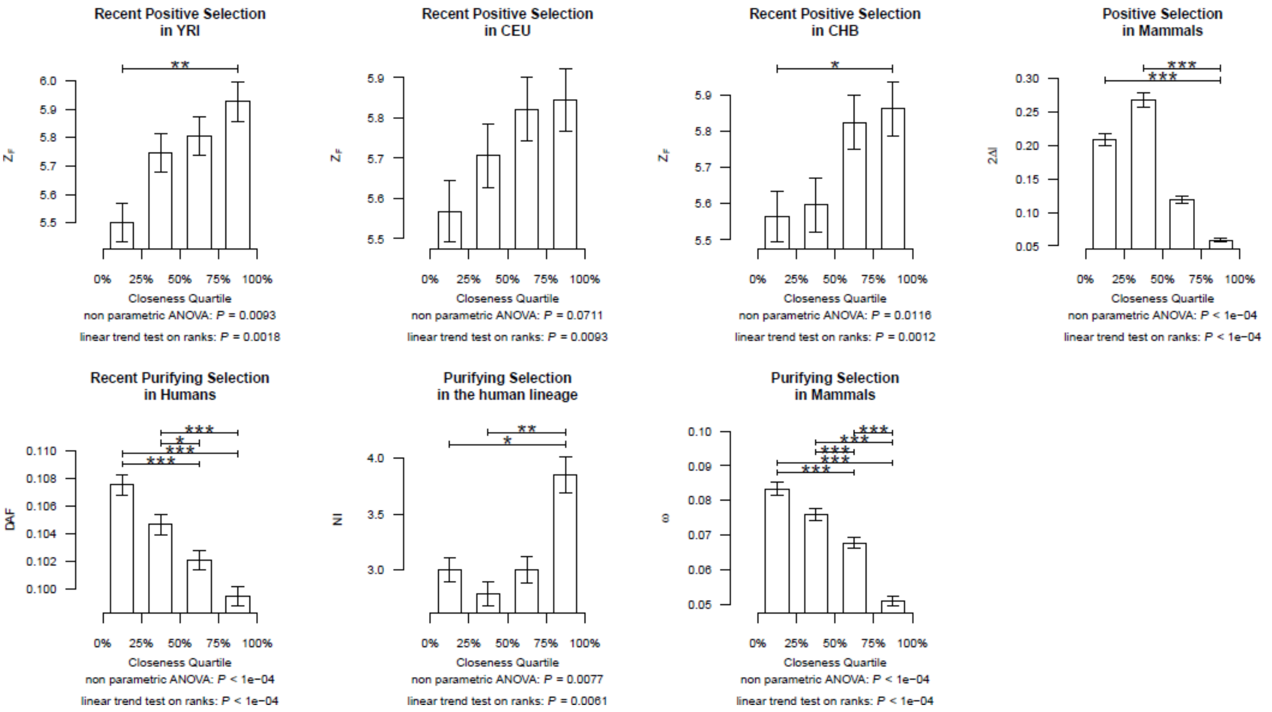

Supplementary Figure 9. Impact of natural selection among groups of genes classified according to their closeness in the BioGRID network.

Genes were classified into four groups according to the closeness quartiles. The median of the positive selection scores ± one median absolute deviation within each group is represented across the *y*-axis. Z_F and $2\Delta\ell$ were used to estimate the likelihood of having been targeted by positive selection in human populations and in mammals, respectively. DAF, NI and ω were used to estimate the impact of purifying selection in recent human populations, in the human lineage and in mammals, respectively. Lower DAF and ω values indicate higher evolutionary constraint estimated from polymorphism and divergence data, respectively, while higher NI values indicate higher evolutionary constraint estimated from both polymorphism and divergence data. A non-parametric ANOVA analysis was performed to contrast whether the medians of the scores are equal across the groups. A trend test on ranks was also carried out to test for a linear relationship between the four quartiles (encoded from 1 to 4) and natural selection scores. A Tukey's honestly significant difference test was further applied to test for all pairwise differences. Significantly different pairs are marked with asterisks. *: $P < 0.05$; **: $P < 0.01$; ***: $P < 0.001$.

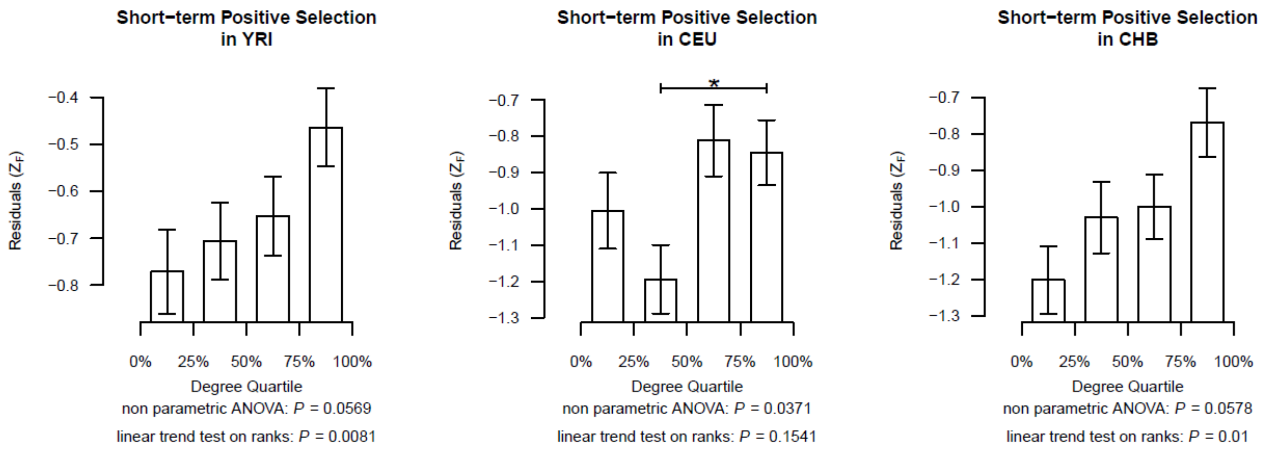

Supplementary Figure 10. Impact of positive selection during recent human evolution among groups of genes classified according to their degree controlling for the effect of purifying selection during mammalian evolution.

In order to test for an association between degree and the Z_F score controlling for purifying selection, we applied a linear regression between Z_F and ω. The linear regression residuals were then used in a Spearman's correlation analysis, a non-parametric ANOVA and a linear trend test on ranks. Genes were classified into four groups according to the degree quartiles. The median of the residuals ± one median absolute deviation within each group is represented across the *y*-axis. A Tukey's honestly significant difference test was further applied to test for all pairwise differences. Significantly different pairs are marked with an asterisk. *: $P < 0.05$; **: $P < 0.01$; ***: $P < 0.001$.

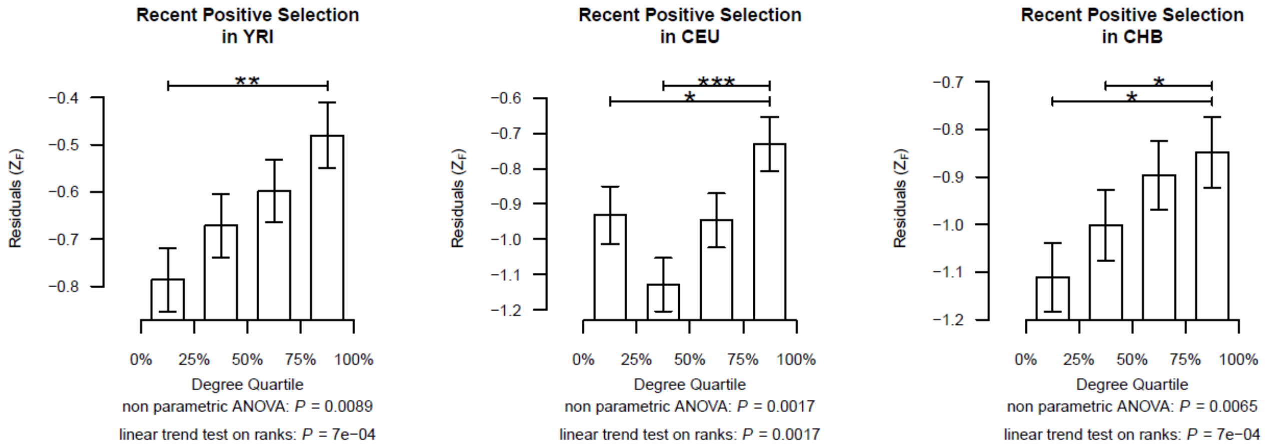

Supplementary Figure 11. Impact of positive selection during human evolution among groups of genes divided according to their degree controlling for covariates of background selection. In order to test for an association between degree and the Z_F scores controlling for background selection, we applied a linear regression between Z_F and both the GC content and the average population-specific recombination rate across the gene. The linear regression residuals were then used in a Spearman's correlation analysis, a non-parametric ANOVA and a linear trend test on ranks. Genes were classified into four groups according to their degree. The median of the residuals ± one median absolute deviation within each group is represented across the *y*-axis. A Tukey's honestly significant difference test was further applied to test for all pairwise differences. Significantly different pairs are marked with asterisks. *: $P < 0.05$; **: $P < 0.01$; ***: $P < 0.001$.
